# Protocol for randomized personalized trial for stress management compared to standard of care

**DOI:** 10.3389/fpsyg.2023.1233884

**Published:** 2023-09-19

**Authors:** Ashley M. Goodwin, Danielle Miller, Stefani D’Angelo, Alexandra Perrin, Ruby Wiener, Brittney Greene, Anne-Marie N. Romain, Lindsay Arader, Thevaa Chandereng, Ying Kuen Cheung, Karina W. Davidson, Mark Butler

**Affiliations:** ^1^Institute of Health System Science, Feinstein Institutes for Medical Research, Northwell Health, New York, NY, United States; ^2^State University of New York at Buffalo, Buffalo, NY, United States; ^3^Gordon F. Derner School of Psychology, Adelphi University, Garden City, NY, United States; ^4^Department of Psychology, St. John’s University, Jamaica, NY, United States; ^5^Mailman School of Public Health, Columbia University, New York, NY, United States; ^6^Donald and Barbara Zucker School of Medicine at Hofstra/Northwell, Northwell Health, Hempstead, NY, United States

**Keywords:** personalized trials, N-of-1, stress management techniques, mindfulness, yoga, physical activity

## Abstract

Stress is a significant public health burden in the United States, with most Americans reporting unhealthy levels of stress. Stress management techniques include various evidence-based treatments shown to be effective but with heterogeneous treatment responses, indicating a lack of uniform benefits for all individuals. Designed to assess a participant’s response to a specific intervention, personalized (N-of-1) trials provide guidance for which treatment (s) work (s) best for the individual. Prior studies examining the effects of mindfulness meditation, yoga, and walking for stress reduction found all three interventions to be associated with significant reductions in self-reported measures of stress. Delivering these treatments using a personalized trial approach has the potential to assist clinicians in identifying the best stress management techniques for individuals with persistently high stress while fostering treatment decisions that consider their personal condition/barriers. This trial will evaluate a personalized approach compared to standard of care for three interventions (guided mindfulness meditation; guided yoga; and guided brisk walking) to manage perceived stress. Participants will respond to daily surveys and wear a Fitbit device for 18 weeks. After a 2-week baseline period, participants in the personalized trial groups will receive 12 weeks of interventions in randomized order, while participants in the standard-of-care group will have access to all interventions for self-directed stress management. After intervention, all participants will undergo 2 weeks of observation, followed by two additional weeks of the stress management intervention of their choosing while continuing outcome measurement. At study completion, all participants will be sent a satisfaction survey. The primary analysis will compare perceived stress levels between the personalized and standard of care arms. The results of this trial will provide further support for the use of personalized designs for managing stress.

**Clinical Trial Registration**: clinicaltrials.gov, NCT05408832.

Protocol version: 9/14/2022, 21-0968-MRB.

## Introduction

Though stress is a prevalent condition worldwide, Americans report stress levels 20% higher than the global average (Heckman, 2022). Americans often report unhealthy levels of stress;

83% of United States workers suffer from work-related stress, and there has been an increase in the prevalence of stress due to life stressors and current events (American Psychological Association, 2019, 2020; Heckman, 2022). Previous research has shown that prolonged stress levels can negatively affect overall mental and physical health (American Psychological Association, 2020). Mindfulness meditation, yoga, and physical activity are common stress management techniques associated with significant reductions in self-reported and/or physiological measures of stress (Goyal et al., 2014; Khoury et al., 2015; Pascoe et al., 2017; Strehli et al., 2021). Yet, these interventions often have high levels of heterogeneity in treatment effects (HTE) for stress reduction, indicating that not all patients receiving a given intervention may benefit equally. Therefore, identifying which of these three treatments may benefit individuals with high levels of stress is difficult. A personalized (N-of-1) trial design, which uses a single-subject experimental approach to evaluate the outcomes of different interventions specific to an individual (Guyatt et al., 1986; Davidson et al., 2021), may be a reasonable approach to effectively treat stress and has been previously recommended in the treatment of depression (Simon and Perlis, 2010; Kronish et al., 2019) and mental health conditions, overall (Insel, 2009).

Previous feasibility pilots have tested remotely implemented personalized trials and found this approach to be feasible and acceptable among interventions for depression, fatigue, and back pain (Kronish et al., 2019; D’Angelo et al., 2022; Butler et al., 2022a,b). In addition to these feasibility trials, evaluation of the effectiveness of a personalized design compared to standard-of-care treatment is needed to determine if changes in treatment, cessation of treatment, or confirmation of the original treatment are needed to improve health outcomes (Guyatt et al., 1990, 2008; Larson, 2010; Joy et al., 2014), and/or if they lead to identification of health outcomes and values important to the patient (Duan et al., 2013).

As stress has high public health burden, high heterogeneity of treatment response, and evidenced-based treatments, it is an ideal candidate for which to test the effectiveness of a personalized trial design. Personalized trials are conducted infrequently in clinical practice (Kravitz et al., 2008; Gabler et al., 2011; Guyatt, 2016), as they are perceived to be overly burdensome (Davidson et al., 2014) and insufficiently appealing to patients or clinicians to justify the cost and effort needed to design and implement them (Kravitz et al., 2008; Guyatt, 2016). However, with the increasing popularity and accessibility of new technologies as well as the ability to process large data sets in a short period of time, personalized trials are now more feasible to scale to clinical practice than ever before.

Few studies have examined the effectiveness of personalized trials to improve clinical outcomes over standard of care (Samuel et al., 2022). As such, testing of this approach is more useful for personalized outcomes uniquely relevant to the values important to the participant (Duan et al., 2013) compared to the standard of care, and it will add to the body of work examining personalized trials. Further, conducting personalized trials is often a decision driven by clinicians or researchers (Bohart et al., 2003; Gabler et al., 2011), with little input from patients. This personalized trial was designed differently; it provides an opportunity to examine the “match” between the treatment informed by the personalized trial data and the treatment selected by an individual patient from the patient’s perspective. This may lead patients to become more involved in clinical decision-making and thus more likely to receive effective treatment to which they will adhere (Entwistle and Watt, 2006; Loh et al., 2007). Because of the return of individualized results to each participant and a planned follow-up period to determine adherence to participant-selected or-preferred stress management, we will be able to analyze how a participant’s personal wellness strategy may be impacted by the customized report received as part of the personalized trial.

The aim of this study is to determine if a personalized trial design will improve stress over standard practice for stress management among 212 participants who self-identify as having elevated stress [Perceived Stress Scale (PSS)] (Cohen et al., 1983). The results from this trial will determine the efficacy of the personalized approach relative to traditional methods for stress management, in which participants choose a stress management technique without randomized data about its effectiveness for them personally.

## Methods

### Study design

**Figure 1:**
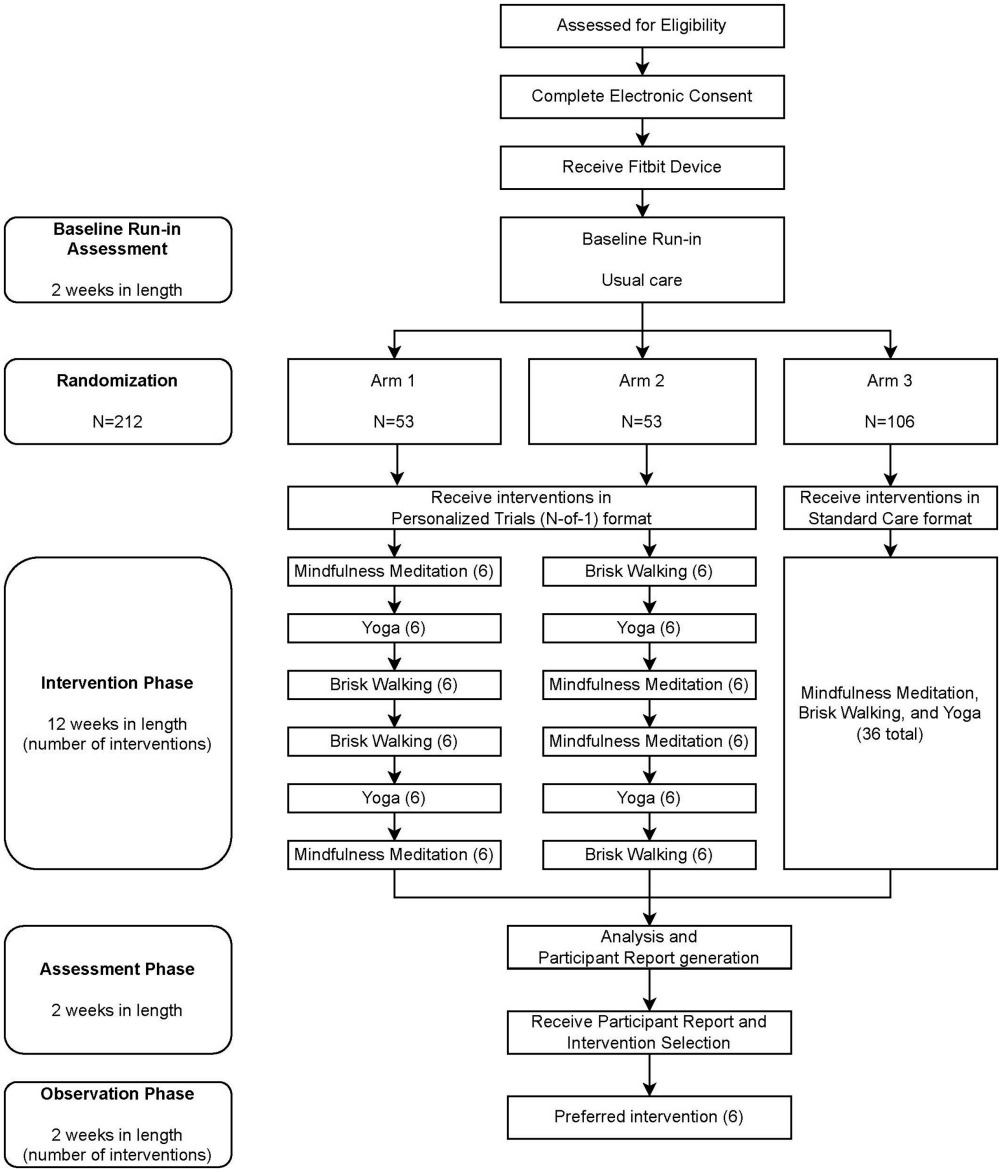
Participant Timeline. Participants in the intervention phase of the personalized trial are encouraged to watch three treatment videos per week (six videos per 2-week treatment block). Participants in the standard of care arm are encouraged to watch 36 videos during the intervention phase but are given no guidance regarding when to watch videos.

A randomized National Institutes of Health (NIH) Stage Model of Behavioral Intervention Stage 2 trial design (Onken et al., 2014) will be used to randomize 212 participants: 106 participants will receive a personalized trial intervention for stress management in one of two possible sequence orders, and 106 participants will receive standard of care. The intervention will be delivered virtually to participants residing in the United States over the course of 18 weeks, divided into a 2-week baseline period, a 12-week intervention period, a 2-week assessment period, and a 2-week post-intervention observation period (see Figure 1). Participants’ levels of stress will be assessed by ecological momentary assessment (EMA) measures. A wearable activity tracker (Fitbit®) will be used to monitor steps continuously and sleep. Intervention components will be delivered by virtual link to an online video or audio recorded by an experienced Zeel wellness provider. Participants will be permitted to use their own Fitbit device (Fitbit Sense™, Fitbit Versa 3™, Fitbit Charge 5™, Fitbit Charge 4™, Fitbit Luxe™, Fitbit Inspire 2™, or similar newer models that are released in the future) or will be provided one (Fitbit Charge 5™) (Haghayegh et al., 2019).

#### Baseline period

The first 2 weeks of the study will be a baseline assessment period. Participants will be asked to engage in their usual methods of managing stress and will be instructed to wear their Fitbit device at all times, including during sleep. Participants will also be asked to rate an EMA of their stress, fatigue, pain, concentration, mood, and confidence three times daily at random times via text message. Each evening, they will answer a survey assessing any stress interventions they completed that day, as well as any side effects they experienced from the stress intervention. Each weekend, participants will complete a longer survey asking them to reflect on their stress over the week. Participants will be asked to wear their Fitbit devices day and night (≥12 h/day), to sync their device with the Fitbit application on their phone at least every 2 days and charge their Fitbit device at least every 4 days. Ten days into the baseline period, study staff will begin to review individual adherence to Fitbit wear and survey responsiveness. Fitbit wear time adherence will be defined as one’s having at least 720 min (12 h) of non-sleep and sleep heart rate activity per day. Survey adherence will be defined as one’s having responded to a survey. Participants that meet at least 80% of the adherence criteria by day 14 will move on to the intervention phase.

#### Intervention period

##### Stress management techniques

Three common stress management techniques will be used: guided mindfulness meditation, guided yoga, and guided brisk walking. Zeel, a commercial wellness technology platform that connects individuals to in-home or in-office services (such as massage and yoga), has been contracted to create recorded guided video and audio content. Recorded video and audio stress management content will be stored on a commercial website, Vimeo. Participants will be provided with a unique link and password to access their own folder of stress management content and will not be asked to provide any personal identifying information on the video website. Data collected will include the date the video was viewed and corresponding view duration. Participants will be able to access intervention videos through smartphone or desktop devices.

After successfully completing the baseline period, participants will be randomized into one of three arms for 12 weeks. Arms 1 and 2 will receive the personalized trial framework and will be prompted to follow a schedule in performing stress management interventions, while participants in Arm 3 will be provided the same interventions without the requirement to follow the established personalized trial framework. During subsequent weeks 15 and 16, participants will be asked to continue to wear their Fitbit and answer study surveys without completing any virtual interventions while their data from baseline and intervention weeks are analyzed and their personalized participant report is generated. Reports for participants in Arms 1 and 2 will include information about which of the three interventions provided the most reduction in momentary stress as well as information about how various other outcomes (e.g., physical activity, sleep, fatigue, and pain) may have changed during weeks the participant was asked to complete each intervention. Reports for participants in Arm 3 will provide details about how stress and other outcomes changed over time but will not provide information about how each of the three interventions may have impacted stress.

##### Post-intervention observation period

Once participants receive their participant reports, regardless of Arm, they will be asked to select an intervention to continue receiving for an additional 2 weeks. Based on their selection, participants will receive another six sessions of stress management content (e.g., if yoga is selected, participants will receive six additional views of the yoga content), however, they will not be prompted to complete the selected intervention. Participants will be observed for 2 weeks and will be asked to continue to wear their Fitbit and answer study surveys. At the end of the 18 weeks, each participant will be provided with a satisfaction survey. Upon completion of data monitoring, participants will be given instructions on how to un-link their Fitbit from the study account.

Additionally, a random sample of 10% of participants from each arm will be asked to participate in a qualitative interview to share their opinions on the participant report. Selected participants will be sent a link to share their availability for a session with a consenting coordinator (s) held via video conference (e.g., Microsoft Teams or a similar program). Interviews will be audio-recorded and transcribed by the study team. On the rare chance that a participant cannot meet via video conference, the interview will be conducted via phone call. The interview will last approximately 60 min. Study recruitment began in May 2022, and enrollment completion is anticipated to occur in August 2023.

### Study population

Healthy individuals 18 years of age and older who self-identify as having a minimum threshold of stress using the PSS (raw score of 20; adapted from NIH Toolbox Perceived Stress Uncorrected T-score, NIH Toolbox 2020) in the last month will be recruited. Inclusion criteria can be found in Table 1. Due to the high prevalence of stress (American Psychological Association, 2019) and a robust recruitment strategy, a large pool of potential participants from which to recruit is anticipated.

**Table 1:** Inclusion/exclusion criteria.

Inclusion criteria	Exclusion criteria
A participant must meet the following criteria to be included in the study: Is aged ≥18 years Speaks English Has a self-report of perceived stress with a raw score of 20 or higher using the PSS Owns and can regularly access a smartphone capable of receiving text messages and accessing the internet Can regularly wear a Fitbit device Lives in the United States	A person who meets the following criteria will be excluded: Are pregnant Does not speak English Does not own or cannot regularly access a smartphone capable of receiving text messages Cannot regularly wear a Fitbit device Is deemed unable to complete the study protocol as a result of cognitive impairment, severe medical or mental illness, or active or prior substance abuse Has planned surgeries 6 months from study start date Has been previously advised by a doctor not to engage in 30 min of brisk walking three times per week Has been previously told by a doctor to not engage in yoga Lives outside the United States

### Recruitment

Participants will be recruited from across the United States. This will include the approximately 83,000 employees of the Northwell Health System (Northwell Health, 2022). Online recruitment of potential participants will include email blasts to Northwell employees, advertisements in newsletters circulated to the Northwell Health team member population, word of mouth, and targeted recruitment emails to individuals who previously expressed interest in personalized trials. To expand beyond the Northwell Health system, individuals will be recruited via outreach to clubs and organizations at local universities alongside advertising and social media posting on Facebook, Instagram, Google, LinkedIn, Reddit, and Craigslist. Given that 93% of United States adults report using the Internet, 69% report ever using Facebook, and 18% reporting using Reddit, online recruitment methods expand the potential span of our outreach (Pew Research Center, 2021). Posting and advertisement will be comprised of multiple formats of information (including videos, images, and text posts).

Interested individuals who respond to any of the above recruitment methods will be directed to a secure, web-based software platform designed to support data capture (REDCap) to read more details about the trial and respond to a screening questionnaire (Harris et al., 2009, 2019). Individuals deemed ineligible for the study based on their responses to the screening questions will be notified immediately within the screening process.

### Consent

Consent will be obtained electronically. Upon completion of screening, eligible individuals will be taken to a view-only version of the electronic consent form on REDCap with a short video explaining details of the study protocol and consent form. They will be notified they can bring questions to the study team at any time and will be provided with a signable version of the electronic consent form, the explanatory video, and a four-question screening measure assessing participant understanding of the protocol and consent process. Individuals who wish to speak with a staff member to clarify details of the study or consent will be given the opportunity to have a 30-min informational phone call with study staff to review key points and ask questions prior to signing the consent form. After the call, study staff will send the eligible participant a link to access a signable version of the electronic consent form, the explanatory video, and a four-question screening measure assessing participant understanding of the protocol and consent process on REDCap (Lawrence et al., 2020). A signed copy of the consent form will be made available to participants. Signed consent and Health Insurance Portability and Accountability Act (HIPAA) forms will be stored electronically on a HIPAA–compliant, Northwell Health–approved shared drive accessible only to approved study staff. The study consent form can be found in the Appendix.

After consenting, participants will be asked to complete an onboarding survey where they will provide information that will allow the trial to be more personalized to their lifestyle, including preferred study start date. Study enrollment will continue until 212 participants have been randomized after the baseline assessment. This study was approved by the Northwell Health Institutional Review Board (IRB).

### Assignment of interventions

Each intervention is assigned a letter as follows: A = Mindfulness Meditation; B = Yoga; and C = Brisk Walking. Of the participants who are enrolled in the study, approximately 53 will be randomized into Arm 1 by the study statistician to receive the protocol in the following order of two-week intervention periods: Mindfulness Meditation, Yoga, Brisk Walking, Brisk Walking, Yoga, and Mindfulness Meditation (ABCCBA). Approximately 53 participants will be randomized into Arm 2 to receive the protocol in the following order of 2-week intervention periods: Brisk Walking, Yoga, Mindfulness Meditation, Mindfulness Meditation, Yoga, and Brisk Walking (CBAABC). Two treatment orders were utilized to help with generating pooled estimates of treatment effects. Approximately 106 participants will be randomized into Arm 3 by the study statistician to receive all three interventions for a 12-week period in no specific order or schedule (standard care). This randomization pattern can be viewed in the participant timeline in Figure 1. Prior to the first participant enrollment, the statistical and data science team will generate a permuted block randomization sequence in 27 blocks of eight participants with pre-specified ratios to ensure equal treatment allocation within each block. This will provide sufficient randomizations for 212 participants with a potential four additional randomizations if necessary. As participants are enrolled, randomizations will be assigned sequentially. Participants, study staff, and statisticians will not be blinded to treatment following assignment.

### Interventions

Once a participant successfully completes baseline and is randomized into the study, they will receive an intervention schedule indicating when they are being asked to complete the guided stress management interventions (Arms 1 and 2) or an intervention schedule indicating they can complete the guided stress management interventions at their own preferred pace (Arm 3). Participants will be instructed to access their intervention video (s) through their unique folder on Vimeo. During intervention weeks, all participants will receive a weekly evening reminder to complete their interventions along with a link and password to access the videos. Participants in Arms 1 and 2 will be able to complete their assigned intervention three times in the Monday-to-Sunday week for a total of 12 times per intervention and 36 total sessions. They will also receive a morning reminder three times per week on their preferred days as indicated in their onboarding survey to complete their interventions along with a link and password to access the video. Participants in Arm 3 will self-directedly be able to complete the three interventions no more than 12 times each for a total of 36 sessions (i.e., the same total amount of intervention sessions as Arm 1 and 2 participants). All participants will be asked to refrain from engaging in other stress management interventions outside their usual regimen for the duration of the study. During all intervention periods, participants will be asked to wear the Fitbit device 24 h a day and answer the four survey measures sent to them via text message daily.

### Adherence

Participant adherence to the protocol will be assessed during the first 14 days of the baseline assessment period. During baseline assessment, study staff will review participant adherence to wearing their Fitbit, EMA measure completion, and survey completion. Participants wearing the Fitbit more than 12 h per day awake and while sleeping will be defined as adherent, as will those who respond to EMA and survey measures. During the 14 days of the baseline period, participants who do not achieve a minimum of 80% adherence to Fitbit wear and study measures will be withdrawn from the study. Participants maintaining 80% adherence or greater will finish out the baseline period and be randomized to the intervention period. Several methods will be used to encourage adherence throughout the study. To promote retention, participants will have short education videos available to them; be provided with protocol reminders via text message; and be encouraged to contact study staff with concerns by phone, email, or secure portal message. Additionally, a regret lottery will be used each week to encourage participation in elements of the personalized trial. All participants in weeks 3–14 of the study are entered in the lottery, but only participants who have achieved 80% adherence for the week on all trial measures (including Fitbit device wear time, EMA measures, and surveys) are eligible to win the $100 prize. All participants, regardless of whether or not they win the lottery, are notified as to whether they achieved 80% adherence that week (Husain et al., 2019).

### Participant report

After the intervention period, participants’ data will be analyzed by statisticians and presented in personalized data reports. The report compares baseline measurements of activity, sleep, concentration, confidence, mood, pain, fatigue, and stress to intervention period measurements. Examples of the personalized reports can be found in the Appendix.

### 2-week additional observation period

After receiving the personalized report, participants will be asked to choose one of the three interventions to continue for an additional 2-week observation period. They are able to complete their preferred intervention six times over these 2 weeks.

### Compensation

Participants who are provided a Fitbit Charge 5™ device will keep their device (a value of $150). During weeks 3–14, participants are entered into a weekly lottery, and winners are awarded a $100 ClinCard (Mastercard), pending adherence to study procedures including wearing the Fitbit device and completing survey measures. A random 10% of participants from each of the three arm sequences will be invited to participate in a qualitative interview following completion of the study to discuss their experiences and will be compensated with a $25 ClinCard for participating in the interview.

### Primary outcome

The primary outcome is the between-arm change in average daily perceived stress, assessed using EMA, 2 weeks post-intervention selection compared to baseline assessment. The EMA used is a measure adapted from the Numeric Pain Rating Scale (Jensen et al., 1999) and has previously been utilized as a self-reported stress measure in other personalized trials (Butler et al., 2022a,b, 2023). This assessment measure is a single-item assessment administered three times daily via text message asking participants to rate their stress (as well as other states described further in “Secondary Outcomes”) in the current moment on a scale of 0–10. The timing of the text messages will be randomized between a participant’s self-reported wake and sleep times. The goal of randomized assessment timing is to get an assessment of participant stress not linked to particular time-points. This method is well supported in EMA literature (Shiffman et al., 2008) and has been implemented with mood disorders in the past (Aan Het Rot et al., 2012). Ratings of 0 indicate no stress at all, with scores of 1–3, 4–6, 7–9, and 10, respectively, indicating a little bit stressed, somewhat stressed, quite a bit stressed, and very much stressed. EMAs are collected daily via surveys using a secure Northwell Health–approved and HIPAA–compliant study platform used for patient engagement and collecting and storing research data. A workflow was constructed for this study to include automated messaging pathways delivered via text message directly to the participant’s smartphone via the platform.

Levels of EMA stress will be aggregated within the baseline assessment period and during the follow-up period to generate an overall mean and standard deviation for each period. The change in EMA stress between baseline and follow-up will be calculated by subtracting these aggregated means. A copy of the EMA can be found in the Appendix.

### Secondary outcomes

The major secondary outcome is proportion of times participants selected the intervention that was recommended to them via their personalized data report. At the end of the intervention in the personalized trial arms (Arm 1 and Arm 2), personalized trial data will be used to identify during which intervention (mindfulness, yoga, or walking) the most stress reduction occurred. This recommendation will be presented in a personalized report sent after completion of the intervention. The number of participants in the personalized trial arms who select this recommended intervention during follow-up relative to the total number of participants in Arms 1 and 2 will be presented as a proportion, with a higher proportion indicating greater levels of agreement. Participants in Arm 3 will similarly be provided with a personalized report and asked to choose a preferred intervention. However, this report will not provide a recommended intervention. Therefore, Arm 3 intervention preference responses are outside of the scope of this outcome.

Other secondary outcomes in the current study will include weekly perceived stress assessed with the PSS-10, modified to be delivered to assess the prior week rather than the prior month. The minimum total score possible is 0 and the maximum total score possible is 40. Higher values represent higher levels of stress. The PSS-10 can be found in the Appendix.

Feasibility will be measured by the mean usability score via the 10-item System Usability Scale (SUS; Brooke, 1996). The SUS is a validated questionnaire that asks users to score each item on a Likert scale from Strongly Disagree (1) to Strongly Agree (5). Individual item scores are multiplied by 2.5 and summed to generate a total score ranging from 0 to 100, with higher scores indicating a greater level of usability. This measure has been utilized and validated in multiple contexts (Brooke, 2013; Lewis, 2018). The SUS will be presented to the participant as addressing the ease of use, complexity, and consistency of the personalized trials system as a whole. It can be found in the Appendix.

Satisfaction will be measured using a 13-item satisfaction survey. The survey will assess participant satisfaction with elements of the trial, including resources like the Fitbit device, the personalized trial design, survey assessment measures, interventions, and the participant report. Participants will be asked to rate their satisfaction on a scale of 1 “Not Very Satisfied” to 5 “Very Satisfied.” The satisfaction survey will also include a seven-item series of questions regarding the participant’s experience in the personalized trial as a whole. Satisfaction with aspects of the trial including the onboarding process and ease of trial adoption will be rated on a scale of 1 “Strongly Disagree” to 5 “Strongly Agree.” Finally, satisfaction with the personalized report will be assessed through five questions on a scale of 1 “Strongly Disagree” to 5 “Strongly Agree.” The final three items on the satisfaction survey include if the participant would recommend the personalized trial to others with stress (i.e., “I would not recommend” to “I would strongly recommend”), how helpful participating in the study was in regard to their symptoms of stress (i.e., “Not at all helpful” to “Extremely helpful”), and a free-text comments box. The satisfaction survey can also be found in the Appendix.

Daily self-reported stress, fatigue, pain, concentration, mood, and confidence ratings will be assessed via EMA using a measure adapted from the Numeric Pain Rating Scale (Jensen et al., 1999). Delivery and assessment of these measures will be identical to the methods used for EMA stress described above. For fatigue, ratings of 0 indicate no feeling of fatigue, with scores of 1–3, 4–6, 7–9, and 10, respectively, indicating a little, somewhat, quite a bit, and very much feeling fatigued. Interpretations of scores remain the same for pain, concentration, and confidence. For mood, ratings of 0 indicate poor mood with scores of 1–3, 4–6, 7–9, and 10, respectively, indicating a fair, good, very good, and excellent mood. Within-subject differences of each EMA rating will be evaluated.

### Other study outcomes

Daily step counts and nightly sleep duration will be assessed by a Fitbit device including Fitbit Charge 5™, Fitbit Sense™, Fitbit Versa 3™, Fitbit Charge 4™, Fitbit Luxe™, Fitbit Inspire 2™ models, or similar newer models that are released in the future. During baseline assessment (2 weeks) and all subsequent study weeks (16 weeks), participants will be asked to wear their Fitbit device each day and night (for a total of 18 weeks overall). Daily step counts and nightly sleep duration will be recorded by the Fitbit device for within-subject differences in daily steps and nightly sleep. Time spent asleep will be calculated using Fitbit’s proprietary method, which has been previously shown to be inferior to polysomnography, though it is potentially useful to individual participants in identifying how each treatment influences their sleep. Participant adherence to the interventions will be measured using the video hosting platform Vimeo. Vimeo records data associated with the viewing of videos on their platform in their Analytics center. For each participant, the intervention adherence rate, defined as unique video views of the appropriate recorded intervention during the assigned interventions, will be captured, calculated, and reported across all applicable individuals with means and standard deviations. For each participant, the follow-up period adherence rate, defined as unique video views of the chosen recorded intervention during the assigned follow-up period, will be calculated and reported across all applicable individuals with means and standard deviations using Vimeo Analytics.

Participant adherence to the surveys via a proportion of survey measures completed (i.e., each EMA, daily, and weekly surveys) will be calculated. Participant adherence to Fitbit-wear via a proportion of days where the Fitbit device was worn will be calculated. Completion rates across all participants will be reported with means and standard deviations. For each participant, the average days of participant-observed sleep data, defined as recorded sleep and wake cycles, will be calculated across all participants with means and standard deviations.

After completion of the trial, a random sample of 10% of participants from each arm will be asked to participate in 60-min qualitative interviews to discuss their experiences. Descriptive content from these recorded and transcribed interviews will be analyzed and reported.

### Heterogeneity of treatment effects

Even in the context of their effectiveness, stress management techniques are ideal for this study’s interventions and personalized framework due in part to the observed high HTE. The within-subject outcomes in the environment of the multiple-crossover approach will allow the effects and impact of each treatment to be examined. Within-subject outcomes will also be assessed for Arm 3 participants, who were not assigned to a multiple-crossover approach and yet will still have the opportunity to partake in multiple interventions. Each of the following measures will have within-subject differences calculated: perceived weekly stress, daily steps, daily sleep, momentary stress, fatigue, pain, mood, concentration, and confidence. Variability in these calculations would be indication of observed HTE for participants in this study.

## Analysis

### Sample size calculation

The sample size for the current trial was calculated based on the primary outcome, which is change in average momentary stress between the baseline and follow-up periods. Estimates of the sample were generated using a two-sample *t*-test comparing the participants in the personalized trial arms vs. the participants in the standard of care arm. With an effect size of Cohen’s *d* = 0.307 identified using pilot data from a previous N-of-1 trial (Butler et al., 2022b) and an alpha level of 0.05, the current trial will achieve 80% power with a sample size of 168 participants (84 in the personalized arms and 84 in the standard of care arm). To ensure sufficient sample size for the primary outcome, we assumed a conservative 20% attrition rate over the course of the trial, yielding a final sample of 212 participants (106 in the personalized arms and 106 in standard of care).

### Primary analysis

To determine whether personalized intervention arms (1 and 2) yield greater reductions in stress relative to standard of care (Arm 3), changes in the primary outcome (EMA stress) will be examined between baseline and follow-up periods first using a two-sample *t*-test. This test will compare mean changes in stress between baseline and follow-up by treatment condition. We hypothesize that participants in the personalized arms will demonstrate greater stress reductions than participants in standard of care. To examine how personalized interventions influenced stress over the duration of the trial, changes in EMA stress over time will be compared between the personalized and standard of care arms using generalized linear mixed models (GLMM) with autoregressive models with order 1 [AR(1)] to account for linear trends between stress ratings over time. Treatment condition (personalized versus standard of care) and week of the trial will be utilized as fixed effects in the analysis and a random effect will be specified for participants.

### Secondary analyses

The proportion of participants in Arms 1 and 2 who selected the treatment recommended for stress reduction in their personalized report with be represented with frequencies and percentages.

Participant responses to the SUS and rating of satisfaction will be reported with means, standard deviations, and frequencies for participant responses for survey items. As the SUS is a standardized measure, overall participant scores will be compared to other comparable digital interventions and to previous digital N-of-1 trials (Butler et al., 2022a,b, 2023).

Additional outcomes including stress measured using PSS-10 and EMA measures of fatigue, pain, mood, confidence, and concentration will be examined using generalized linear mixed models (GLMM) with autoregressive models with order 1 (AR(1)) to account for linear trends between stress ratings over time. As with analyses of the primary outcome, time (in weeks) and treatment condition will be set as fixed effect while participant will be used as a random effect in the model. These analyses will examine the effects personalized treatment and time on each outcome while accounting for individual differences in participants.

Further, we will conduct regression analyses for all trial outcomes using generalized linear mixed models (GLMM) with autoregressive models with order 1 [AR(1)] for each participant on each outcome. These regression models will be utilized to identify the effect of each treatment on each outcome for every individual participant accounting for changes in the outcome variable over time. Results from these regressions will be presented to participants in a participant report delivered after trial completion. The methods used will be identical to previously published personalized N-of-1 trials (Butler et al., 2022a,b, 2023).

### Heterogeneity of treatment effects

To examine the utility of conducting a personalized trial for treatment of stress, we will examine the HTE with the personalized trial Arms 1 and 2 using methods previously published (Cheung and Mitsumoto, 2022). This will involve modeling the effects of mindfulness, yoga, and brisk walking relative to each other on all outcomes using linear mixed models with first-order autocorrelation [AR(1)]. We will then compare two models (one with a random slope and one with a random intercept) on the effect of treatment on each outcome using a likelihood ratio test. The random slope model accounts for HTE, whereas the random intercept model does not. If this likelihood ratio test is found to be statistically significant (value of *p* < 0.05), it would support the interpretation that HTE exists for a particular outcome. To further identify whether a personalized trial would be helpful in examining the effects of mindfulness, yoga, and brisk walking treatments for each of the outcomes assessed, we calculated an index of heterogeneity to quantify the HTE for each treatment on each outcome (Cheung, 2022; Cheung and Mitsumoto, 2022). This index measures the extent of HTE by incorporating the within-participant variation, between-participant variation, and average of the treatment effects into the standard deviations of the random slope. Under special conditions for personalized trials, this index is equivalent to the statistical power of stating that personalized trials are superior to non–personalized trial alternatives in a particular sample of participants. Higher values for this index will suggest that future trials evaluating the effect of this treatment on stress may want to utilize a personalized design.

## Discussion

The current study represents a significant opportunity to evaluate the feasibility and effectiveness of personalized (N-of-1) trials for stress management. Steady increases in stress reported among Americans today at rates higher than the worldwide average, exacerbated by recent current events, highlight the urgency of this condition as a public health burden (American Psychological Association, 2019, 2020). Various evidenced-based treatments for stress management have been shown to be effective at reducing self-reported stress, though not all patients benefit from these techniques due to a heterogeneity of treatment effects. One reason for this difference in treatment outcomes may be because the gold standard for research trials is the randomized controlled trial (RCT) that uses a between-subjects design with limited variation within the treatment. Participants are randomized to a single treatment and conclusions can only be drawn about the hypothetical average participant (Greenfield et al., 2007), underscoring a flawed assumption that between-subject treatment change is comparable to within-subject treatment change (Davidson et al., 2014). Still, clinicians rely on evidence from RCTs as the standard of care to determine the optimal treatment for a particular patient. When individuals respond differently than this “average” participant from these trials, treatment benefits are unrealized or may result in side effects or unintended consequences. As such, research designs to help patients and their clinicians make better health-related decisions are needed to guide treatment selection that will maximize effectiveness of the treatment, and stress is an ideal use case to test this approach.

Further, the current trial will advance the goals of personalized medicine. The central idea of personalized care in medicine is that personal characteristics can help clinicians tailor treatment decisions to maximize efficacy (Schneider et al., 2015). In research design, this same approach would use a participant’s own data to determine treatment intervention. The personalized treatment arms for stress management will provide direct, objective evidence about how useful a particular intervention is for a specific individual. This personalized approach is akin to a multiple crossover trial whose purpose is to identify the optimal treatment for a single patient (Guyatt et al., 1986; Sedgwick, 2012), but without confounding by covariates that may emerge in between-subject trials. Further, the individualized feedback afforded to each participant in their personalized report may foster greater participation in selecting their treatment regimen among the evidence-base strategies, an empowerment that is considered crucial to patient engagement and delivery of patient-centered care (Nikles et al., 2005; Shamseer et al., 2016). For example, of 71 personalized trials for patients with any chronic pain, 46 patients (65%) opted to change their pain medication due to trial results (Nikles et al., 2005).

Wearable technology and a remote delivery platform will be used to lessen the perceived burden of personalized trials, enabling highly standardized delivery and wider dissemination of intervention components. Ultimately, this study seeks to determine how well personalized (N-of-1) trial designs provide the framework for matching patient with treatment, which in turn will provide clinicians and researchers with the insights needed to improve treatment strategies for stress. This trial will also assess the effectiveness of personalized trials to improve clinical outcomes, which has seldom been done in prior research (Samuel et al., 2022).

As clinicians traditionally have relied on evidence from RCTs to determine a treatment for individual patients, who often respond differently than the hypothetical average patient in these trial designs (Greenfield et al., 2007), the results of this study examining the effectiveness of stress treatments delivered utilizing the personalized trial approach may provide clinicians with the best stress management techniques to monitor and make treatment decisions for patients with chronic stress.

The findings from this study may also generalize to other sectors of research and clinical practice by determining the effectiveness of the personalized design to select the best treatment for an individual patient. For example, researchers have suggested a strategic plan to improve public health via personalized care based on individual responses in areas of life affecting mental health, such as anxiety and depression (Insel, 2009; Simon and Perlis, 2010). Other areas of research and medicine ripe for investigation using the personalized design to determine individualized treatments include diabetes, irritable bowel syndrome, asthma, and insomnia (Samuel et al., 2022). There is also potential for using personalized designs among patients with multimorbidity, in which the evidence drawn from conventional RCTs is particularly vulnerable to clinical guidance reliant on treatments found to be safe and effective for the average patient and specific to treatment of a single condition (Suls et al., 2022). In fact, in a recent online survey conducted among United States patients with two or more prespecified personalized trial–amenable chronic conditions, 82% reported being interested in participating in personalized trials (Derby et al., 2021).

Despite the potential benefits of the current trial, there are also some potential limitations. Firstly, we do not collect comorbidity information (e.g., physical limitations, medical comorbidity, and psychological diagnoses), which may affect the usefulness of each of our intervention methods. However, as the goal of the trial is to use personalized methods to identify the most effective intervention on an individual level, we hope our personalized methods will allow participants to identify which intervention works best for them regardless of what limitations exist in their lives. Secondly, the current trial does not adjust for carryover effects. It is possible that the effects of yoga may carryover to brisk walking or mindfulness treatment periods. While the autoregressive models utilized in the current trial can help to mitigate some of these issues, it is possible that the length and magnitude of carryover effects may vary significantly between participants.

Aligned with a new vision for clinical science and intervention development conceptualized by the NIH Stage Model of Behavioral Intervention, and with the shared goal of establishing interventions that promote the physical and mental health of individuals (Onken et al., 2014), this study offers evaluation of an approach for conducting trials where personalized data derived from wearable technology and engagement with the virtual trial components are used to inform treatment selection for individuals experiencing stress. Similarly, results from this study will offer a foundation for the development of larger personalized trials, in stress and other conditions, where improving the effectiveness of already-established evidence-based treatments is possible by matching people to the best treatment for their own characteristics.

## Ethics statement

The studies involving humans were approved by Northwell Health Institutional Review Board. The studies were conducted in accordance with the local legislation and institutional requirements. The participants provided their written informed consent to participate in this study.

## Author contributions

MB, SD’A, DM, YK, and KD contributed to conception and design of the study and development of the study protocol. TC and YK will perform the statistical analyses. AG and DM wrote the first draft of the manuscript. SD’A, AP, BG, RW, A-MR, and LA wrote sections of the manuscript. All authors contributed to the article and approved the submitted version.

## Funding

This work was supported by the National Library of Medicine of the National Institutes of Health (grant number R01LM012836), PCORI (ME-1403-12304). The funders had no role in the design and conduct of the study; collection, management, analysis, and interpretation of the data; preparation, review, or approval of the manuscript; or decision to submit the manuscript for publication.

## Conflict of interest

The authors declare that the research was conducted in the absence of any commercial or financial relationships that could be construed as a potential conflict of interest.

## Publisher’s note

All claims expressed in this article are solely those of the authors and do not necessarily represent those of their affiliated organizations, or those of the publisher, the editors and the reviewers. Any product that may be evaluated in this article, or claim that may be made by its manufacturer, is not guaranteed or endorsed by the publisher.

## Author disclaimer

The views expressed in this paper are those of the authors and do not represent the views of the National Institutes of Health, the U.S. Department of Health and Human Services, or any other government entity.

## References

[ref1] Aan Het Rot M. Hogenelst K. Schoevers R. A. (2012). Mood disorders in everyday life: A systematic review of experience sampling and ecological momentary assessment studies. Clin. Psychol. Rev. 32, 510–523. doi: 10.1016/j.cpr.2012.05.007 22721999

[ref2] American Psychological Association (2019). Stress in America: Stress and current events. Stress in America™ Survey.

[ref3] American Psychological Association (2020). 10 top trends for 2020. Monitor on Psychology 51. Available at: https://www.apa.org/monitor/2020/01/cover-trends (Accessed March 20, 2023).

[ref4] Bohart A. C. O’hara M. M. Leitner L. M. Wertz F. Stern E. M. Schneider K. . (2003). Recommended principles and practices for the provision of humanistic psychosocial services: Alternative to mandated practice and treatment guidelines. (Accessed November, 1, 2005).

[ref5] Brooke J. (1996). “SUS-a quick and dirty usability scale,” in Usability evaluation in industry. Eds. Jordan P. W. Thomas B. Weerdmeester B. A. McClelland A. L. (Taylor & Francis), 189–194.

[ref6] Brooke J. (2013). SUS: a retrospective. J. Usability Stud. 8, 29–40. doi: 10.5555/2817912.2817913

[ref7] Butler M. D’Angelo S. Kaplan M. Tashnim Z. Miller D. Ahn H. . (2022b). A series of virtual interventions for chronic lower back pain: a feasibility pilot study for a series of personalized (N-of-1) trials. Harvard Data Sci. Rev. 4. doi: 10.1162/99608f92.72cd8432 PMC10443938 37609556

[ref8] Butler M. D’Angelo S. Perrin A. Rodillas J. Miller D. Arader L. . (2023). A series of virtual melatonin supplement interventions for poor sleep: a feasibility pilot study protocol for a series of personalized (N-of-1) trials. JMIR Res. Protoc. 12:e45313. doi: 10.2196/45313, PMID: 37535419 PMC10436115

[ref9] Butler M. D'Angelo S. Lewis C. Miller D. Perrin A. Suls J. . (2022a). Series of virtual light therapy interventions for fatigue: a feasibility pilot study protocol for a series of personalised (N-of-1) trials. BMJ Open 12:e055518. doi: 10.1136/bmjopen-2021-055518, PMID: 36283748 PMC9608534

[ref10] Cheung YK (2022). Personalized (N-of-1) trial design tools. Available at: https://roadmap2health.io/cmi/

[ref11] Cheung K. Mitsumoto H. (2022). Evaluating personalized (N-of-1) trials in rare diseases: how much experimentation is enough? Harvard Data Sci. Rev. doi: 10.1162/99608f92.e11adff0 PMC10813653 38283317

[ref12] Cohen S. Kamarck T. Mermelstein R. (1983). Perceived Stress Scale [Database record]. APA PsycTests.

[ref13] D’Angelo S. Miller D. Monane R. (2022). Personalized feedback for personalized trials: construction of summary reports for participants in a series of personalized trials for chronic lower back pain. Harvard Data Sci. Rev. doi: 10.1162/99608f92.d5b57784 PMC10673635 38009134

[ref14] Davidson K. W. Peacock J. Kronish I. M. Edmondson D. (2014). Personalizing behavioral interventions through single-patient (N-of-1) trials. Soc. Personal. Psychol. Compass 8, 408–421. doi: 10.1111/spc3.12121, PMID: 25267928 PMC4175746

[ref15] Davidson K. W. Silverstein M. Cheung K. Paluch R. A. Epstein L. H. (2021). Experimental designs to optimize treatments for individuals: personalized N-of-1 trials. JAMA Pediatr. 175, 404–409. doi: 10.1001/jamapediatrics.2020.5801, PMID: 33587109 PMC8351788

[ref16] Derby L. Kronish I. M. Wood D. Cheung Y. K. K. Cohn E. Duan N. . (2021). Using a multistakeholder collaboratory and patient surveys to inform the conduct of personalized (N-of-1) trials. Health Psychol. 40, 230–241. doi: 10.1037/hea0001058, PMID: 33856830

[ref17] Duan N. Kravitz R. L. Schmid C. H. (2013). Single-patient (n-of-1) trials: a pragmatic clinical decision methodology for patient-centered comparative effectiveness research. J. Clin. Epidemiol. 66, S21–S28. doi: 10.1016/j.jclinepi.2013.04.006, PMID: 23849149 PMC3972259

[ref18] Entwistle V. A. Watt I. S. (2006). Patient involvement in treatment decision-making: the case for a broader conceptual framework. Patient Educ. Couns. 63, 268–278. doi: 10.1016/j.pec.2006.05.002, PMID: 16875797

[ref19] Gabler N. B. Duan N. Vohra S. Kravitz R. L. (2011). N-of-1 trials in the medical literature: a systematic review. Med. Care 49, 761–768. doi: 10.1097/MLR.0b013e318215d90d 21478771

[ref20] Goyal M. Singh S. Sibinga E. M. Gould N. F. Rowland-Seymour A. Sharma R. . (2014). Meditation programs for psychological stress and well-being: a systematic review and meta-analysis. JAMA Intern. Med. 174, 357–368. doi: 10.1001/jamainternmed.2013.13018, PMID: 24395196 PMC4142584

[ref21] Greenfield S. Kravitz R. Duan N. Kaplan S. H. (2007). Heterogeneity of treatment effects: implications for guidelines, payment, and quality assessment. Am. J. Med. 120, S3–S9. doi: 10.1016/j.amjmed.2007.02.002, PMID: 17403380

[ref22] Guyatt G. (2016). N of 1 randomized trials: a commentary. J. Clin. Epidemiol. 76, 4–5. doi: 10.1016/j.jclinepi.2015.09.020 27063206

[ref23] Guyatt G. Jaeschke R. McGinn T. Rennie D. Meade M. Cook D. (2008). “N-of-1 randomized controlled trials: study design” in Users’ Guides to the Medical Literature (United States: American Medical Association), 179–192.

[ref24] Guyatt G. H. Keller J. L. Jaeschke R. Rosenbloom D. Adachi J. D. Newhouse M. T. (1990). The n-of-1 randomized controlled trial: clinical usefulness: our three-year experience. Ann. Intern. Med. 112, 293–299. doi: 10.7326/0003-4819-112-4-293 2297206

[ref25] Guyatt G. Sackett D. Taylor D. W. Ghong J. Roberts R. Pugsley S. (1986). Determining optimal therapy—randomized trials in individual patients. N. Engl. J. Med. 314, 889–892. doi: 10.1056/NEJM198604033141406 2936958

[ref26] Haghayegh S. Khoshnevis S. Smolensky M. H. Diller K. R. Castriotta R. J. (2019). Accuracy of wristband Fitbit models in assessing sleep: systematic review and meta-analysis. J. Med. Internet Res. 21:e16273. doi: 10.2196/16273, PMID: 31778122 PMC6908975

[ref27] Harris P. A. Taylor R. Minor B. L. Elliott V. Fernandez M. O'Neal L. . (2019). The REDCap consortium: building an international community of software platform partners. J. Biomed. Inform. 95:103208. doi: 10.1016/j.jbi.2019.103208, PMID: 31078660 PMC7254481

[ref28] Harris P. A. Taylor R. Thielke R. Payne J. Gonzalez N. Conde J. G. (2009). Research electronic data capture (REDCap)—a metadata-driven methodology and workflow process for providing translational research informatics support. J. Biomed. Inform. 42, 377–381. doi: 10.1016/j.jbi.2008.08.010, PMID: 18929686 PMC2700030

[ref29] Heckman W. (2022) 42 worrying Workplace Stress Statistics. The American Institute of Stress. Available at: https://www.stress.org/42-worrying-workplace-stress-statistics (Accessed March 20, 2023).

[ref30] Husain S. A. Diaz K. M. Schwartz J. E. Parsons F. E. Burg M. M. Davidson K. W. . (2019). Behavioral economics implementation: regret lottery improves mHealth patient study adherence. Contemp. Clin. Trials Commun. 15:100387. doi: 10.1016/j.conctc.2019.100387, PMID: 31198881 PMC6555893

[ref31] Insel T. R. (2009). Translating scientific opportunity into public health impact: a strategic plan for research on mental illness. Arch. Gen. Psychiatry 66, 128–133. doi: 10.1001/archgenpsychiatry.2008.540 19188534

[ref32] Jensen M. P. Turner J. A. Romano J. M. Fisher L. D. (1999). Comparative reliability and validity of chronic pain intensity measures. Pain 83, 157–162. doi: 10.1016/S0304-3959(99)00101-3 10534586

[ref33] Joy T. R. Monjed A. Zou G. Y. Hegele R. A. McDonald C. G. Mahon J. L. (2014). N-of-1 (single-patient) trials for statin-related myalgia. Ann. Intern. Med. 160, 301–310. doi: 10.7326/M13-1921, PMID: 24737272

[ref34] Khoury B. Sharma M. Rush S. E. Fournier C. (2015). Mindfulness-based stress reduction for healthy individuals: a meta-analysis. J. Psychosom. Res. 78, 519–528. doi: 10.1016/j.jpsychores.2015.03.009, PMID: 25818837

[ref35] Kravitz R. L. Duan N. Niedzinski E. J. Hay M. C. Subramanian S. K. Weisner T. S. (2008). What ever happened to N-of-1 trials? Insiders' perspectives and a look to the future. Milbank Q. 86, 533–555. doi: 10.1111/j.1468-0009.2008.00533.x, PMID: 19120979 PMC2690377

[ref36] Kronish I. M. Cheung Y. K. Julian J. Parsons F. Lee J. Yoon S. . (2019). Clinical usefulness of bright white light therapy for depressive symptoms in cancer survivors: Results from a series of personalized (n-of-1) trials. Health 8:10. doi: 10.3390/healthcare8010010 PMC7151038 31905890

[ref37] Larson E. B. (2010). N-of-1 trials: a new future? J. Gen. Intern. Med. 25, 891–892. doi: 10.1007/s11606-010-1440-8, PMID: 20632123 PMC2917674

[ref38] Lawrence C. E. Dunkel L. McEver M. Israel T. Taylor R. Chiriboga G. . (2020). A REDCap-based model for electronic consent (eConsent): moving toward a more personalized consent. J. Clin. Transl. Sci. 4, 345–353. doi: 10.1017/cts.2020.30, PMID: 33244416 PMC7681162

[ref39] Lewis J. R. (2018). The system usability scale: past, present, and future. Int. J. Hum. Comp. Interact. 34, 577–590. doi: 10.1080/10447318.2018.1455307

[ref40] Loh A. Leonhart R. Wills C. E. Simon D. Härter M. (2007). The impact of patient participation on adherence and clinical outcome in primary care of depression. Patient Educ. Couns. 65, 69–78. doi: 10.1016/j.pec.2006.05.007, PMID: 17141112

[ref41] Nikles C. J. Clavarino A. M. Del Mar C. B. (2005). Using n-of-1 trials as a clinical tool to improve prescribing. Br. J. Gen. Pract. 55, 175–180. PMID: 15808031 PMC1463086

[ref42] Northwell Health (2022). About Northwell Health. About Northwell. Available at: https://www.northwell.edu/about-northwell (Accessed March 27, 2023)

[ref43] Onken L. S. Carroll K. M. Shoham V. Cuthbert B. N. Riddle M. (2014). Reenvisioning clinical science: unifying the discipline to improve the public health. Clin. Psychol. Sci. 2, 22–34. doi: 10.1177/2167702613497932, PMID: 25821658 PMC4374633

[ref44] Pascoe M. C. Thompson D. R. Ski C. F. (2017). Yoga, mindfulness-based stress reduction and stress-related physiological measures: a meta-analysis. Psychoneuroendocrinology 86, 152–168. doi: 10.1016/j.psyneuen.2017.08.008, PMID: 28963884

[ref45] Pew Research Center (2021). Internet/Broadband Fact Sheet. Available at: https://www.pewresearch.org/internet/fact-sheet/internet-broadband/https://www.pewresearch.org/internet/fact-sheet/internet-broadband/

[ref46] Samuel J. P. Wootton S. H. Holder T. Molony D. (2022). A scoping review of randomized trials assessing the impact of n-of-1 trials on clinical outcomes. PLoS One 17:e0269387. doi: 10.1371/journal.pone.0269387, PMID: 35653405 PMC9162303

[ref47] Schneider R. L. Arch J. J. Wolitzky-Taylor K. B. (2015). The state of personalized treatment for anxiety disorders: a systematic review of treatment moderators. Clin. Psychol. Rev. 38, 39–54. doi: 10.1016/j.cpr.2015.02.004 25795293

[ref48] Sedgwick P. (2012). N of 1 trials. BMJ Clin. Res. 344:e844. doi: 10.1136/bmj.e844

[ref49] Shamseer L. Sampson M. Bukutu C. Schmid C. H. Nikles J. Tate R. . (2016). CONSORT extension for reporting N-of-1 trials (CENT) 2015: Explanation and elaboration. J. Clin. Epidemiol. 76, 18–46. doi: 10.1016/j.jclinepi.2015.05.018, PMID: 26272791

[ref50] Shiffman S. Stone A. A. Hufford M. R. (2008). Ecological momentary assessment. Annu. Rev. Clin. Psychol. 4, 1–32. doi: 10.1146/annurev.clinpsy.3.022806.091415 18509902

[ref51] Simon G. E. Perlis R. H. (2010). Personalized medicine for depression: can we match patients with treatments? Am. J. Psychiatr. 167, 1445–1455. doi: 10.1176/appi.ajp.2010.09111680, PMID: 20843873 PMC3723328

[ref52] Strehli I. Burns R. D. Bai Y. Ziegenfuss D. H. Block M. E. Brusseau T. A. (2021). Mind–body physical activity interventions and stress-related physiological markers in educational settings: A systematic review and meta-analysis. Int. J. Environ. Res. Public Health 18:224. doi: 10.3390/ijerph18010224 PMC7795448 33396730

[ref53] Suls J. Alfano C. Yap C. (2022). Personalized (N-of-1) trials for patient-centered treatments of multimorbidity. Harvard Data Science Review. 10.1162/99608f92.d99e6ff5 PMC10673634 38009131

