# Supplementary material for: Protocol for randomized personalized trial for stress management compared to standard of care
Source: Front Psychol. 2023 Sep 19;14:1233884. doi: 10.3389/fpsyg.2023.1233884 (PMC10546313; doi:10.3389/fpsyg.2023.1233884)
Supplement: Supplementary file 1 [file Data_Sheet_1.PDF]

Personalized Trial for Stress Management

Your Stress Therapy Results

TestID_01

12/01/2021 - 12/08/2021

NOTE: This report is not meant to offer medical advice.The goal of this report is to help you understand how certain interventions may impact your self−reported stress. You should seek a medical professional's opinion if you have any questions or concerns.

Feinstein Institutes for Medical Research
Northwell Health

This study was made possible by NIH (R01LM012836) and PCORI (ME-1403-12304).

Visual Summary

Our study team has determined that the most reliable measure of overall stress is momentary stress. This was measured through the three daily Ecological Momentary Assessments (the check-in surveys) you completed during the trial. This page of your report shares which intervention **best improved** your **momentary stress levels**, plus other health benefits of the interventions.

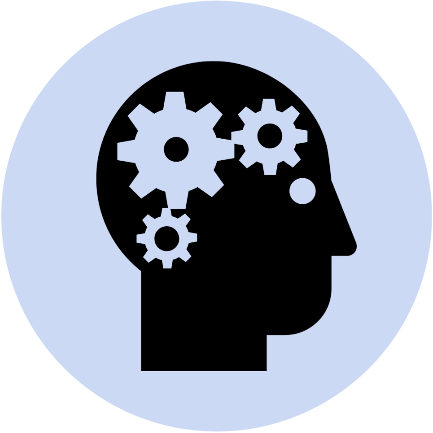

Mindfulness meditation helped your stress levels best!

During mindfulness meditation weeks, you reported the most **improved momentary stress** measured in the moment relative to baseline:

- This improvement was greater than the improvements from yoga or walking.
- This improvement was statistically significant.

Compared to baseline, mindfulness meditation also improved your:

- ✓ Steps*
- ✓ Sleep Duration*
- ✓ Weekly Activity*
- ✓ Sleep Efficiency
- ✓ Average Minutes Awake
- ✓ Concentration*
- ✓ Confidence*
- ✓ Mood
- ✓ Pain*
- ✓ Fatigue

Other data presented in this report give information about other impacts of all of the interventions. These may be helpful in you drawing conclusions about wellbeing techniques that are beneficial **to you**. We encourage you to read the **whole report**, and to pay attention to all benefits and drawbacks presented to you.

Please see the "Visual Summary" video for an explanation of this page.

An asterisk (*) next to a result means the result was statistically significant.

Fitbit Averages

Compared to **baseline** weeks, you had a *decrease* in sleep duration and activity* and an *increase* in steps* during yoga weeks.

Compared to **baseline** weeks, you had an *increase* in steps*, sleep duration*, and activity* during **mindfulness meditation** weeks.

Compared to **baseline** weeks, you had a *decrease* in steps and an *increase* in sleep duration* and activity* during **brisk walk** weeks.

	Daily Steps	Nightly Sleep	Weekly Activity
Baseline	4,936 steps	306 minutes	200 minutes
Yoga	↑ +3,000 steps	↓ −1 minute	↓ −110 minutes
Mindfulness	↑ +3,000 steps	↑ +3 minutes	↑ +20 minutes
Brisk Walk	↓ −110 steps	↑ +3 minutes	↑ +55 minutes

Please see the "Fitbit Averages" video for an explanation of this page.

An asterisk (*) next to a result means the result was statistically significant.

Daily Momentary Assessment

Momentary Stress

Each day at 3 random times you were asked to rate your stress in that moment, which all together is your overall *momentary stress*. Experiencing stress levels in the “Not at All” to “A Little Bit” range is ideal, and is reflected by the bar charts below. The donut charts above the bar charts display the total percent of the intervention you completed.

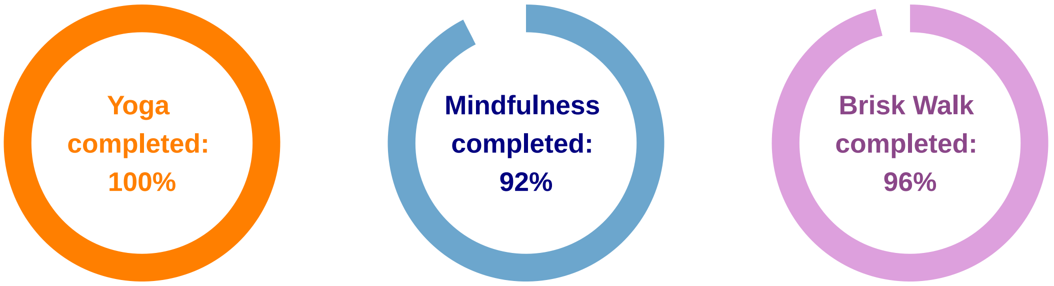

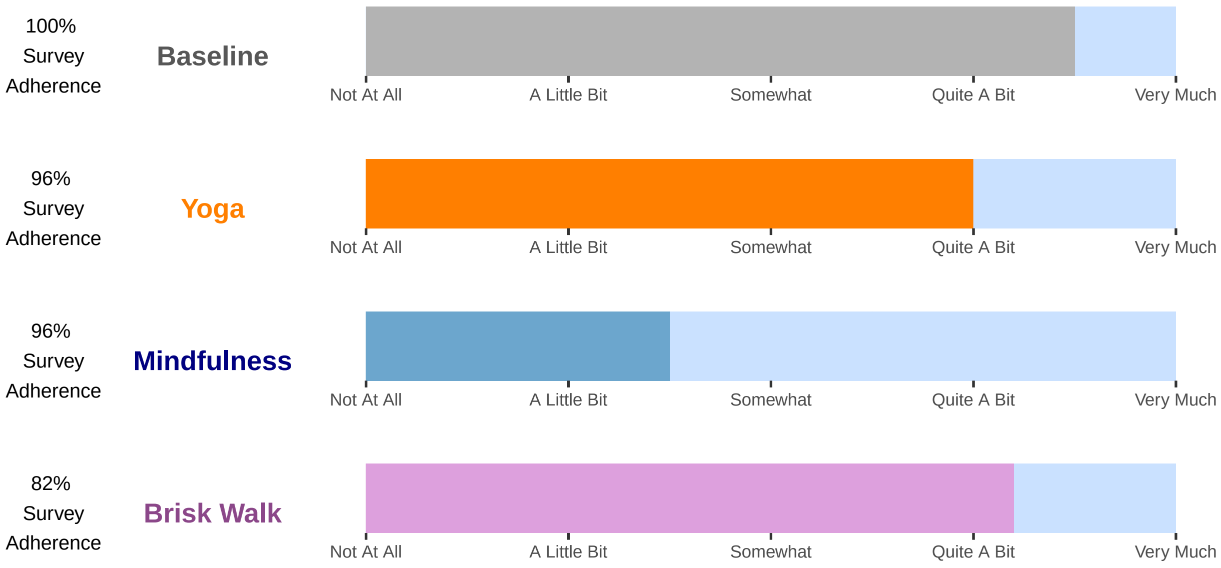

You reported *more* momentary stress during yoga weeks than during your **baseline**.

You reported *less* momentary stress during mindfulness weeks than during your **baseline**.

You reported *more* momentary stress during brisk walking weeks than during your **baseline**.

Please see the "Daily Assessment" video for an explanation of this page.

An asterisk (*) next to a result means the result was statistically significant.

Side Effects

You reported experiencing side effects 5 days of the study. The treatment that you experienced the least number of days with side effects during is ideal.

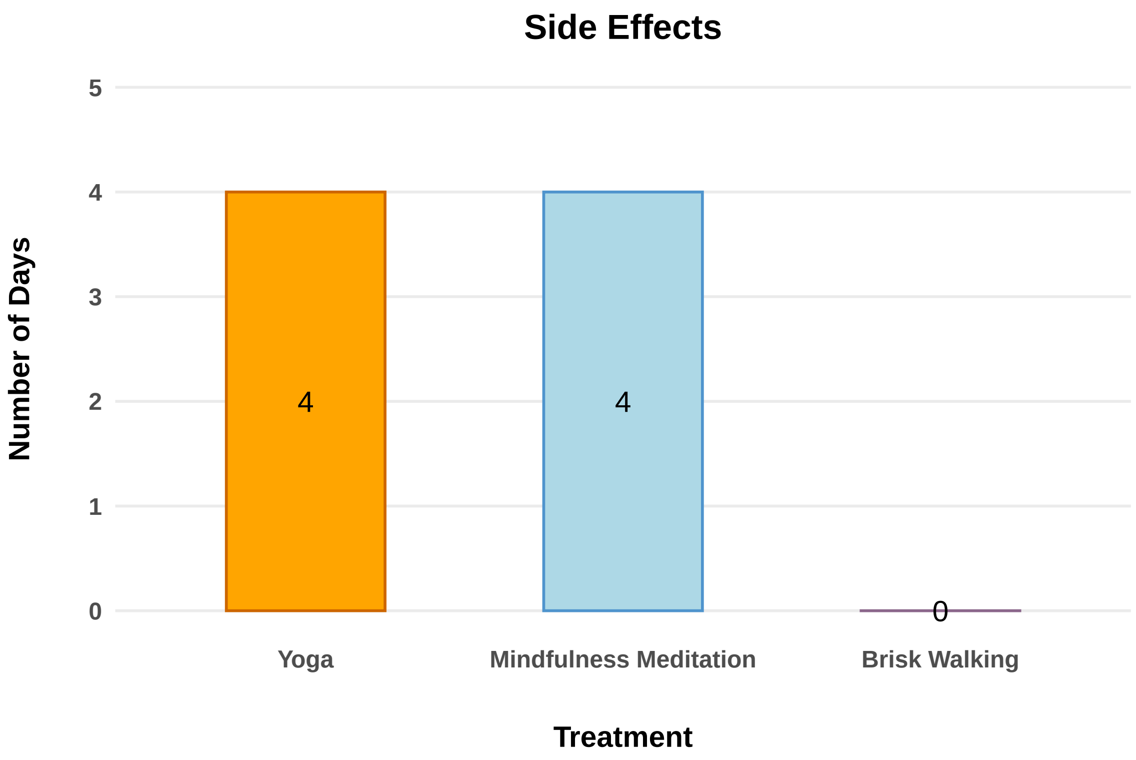

You reported the *least* number of days with side effects during **brisk walking**.

Please see the "Daily Assessment" video for an explanation of this page.

An asterisk (*) next to a result means the result was statistically significant.

Sleep Hygiene

Sleep Efficiency

Sleep Efficiency is based on the total time in bed actually spent sleeping, and is a percentage. Sleep efficiency that is greater than or equal to 85% is an indicator of good sleep quality[3].

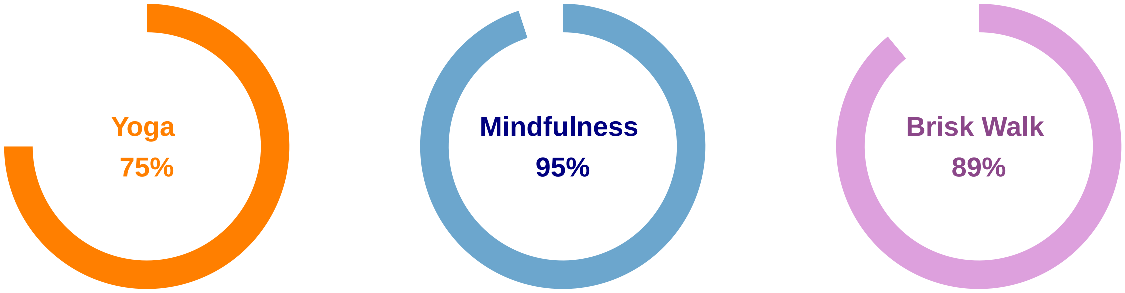

You experienced the *most* efficient sleep while completing **mindfulness** sessions.

Average Minutes Awake

Average minutes awake is the average number of minutes you spent awake each night after falling asleep according to your Fitbit. *Less* minutes awake throughout the night is ideal.

Yoga | Mindfulness | Brisk Walk

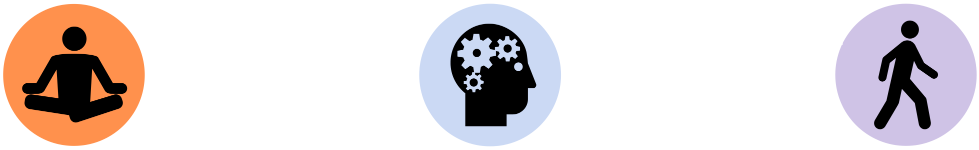

34 minutes | 18 minutes | 24 minutes

You spent the *least* average minutes awake at night while completing **mindfulness** sessions.

Please see the "Sleep Hygiene" video for an explanation of this page.

An asterisk (*) next to a result means the result was statistically significant.

Momentary Patterns

Every day at three random times you were asked to rate your momentary state in that moment, which all together are your *momentary patterns*.

STRESS

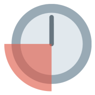

Your highest average stress levels were from 6 PM to 9 PM during brisk walk weeks

STRESS

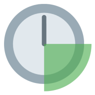

Your lowest average stress levels were from 3 PM to 6 PM during yoga weeks

CONCENTRATION

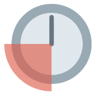

Your lowest average concentration levels were from 6 PM to 9 PM during mindfulness weeks

CONCENTRATION

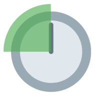

Your highest average concentration levels were from 9 AM to 12 PM during yoga weeks

CONFIDENCE

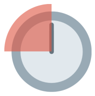

Your lowest average confidence levels were from 9 AM to 12 PM during mindfulness weeks

CONFIDENCE

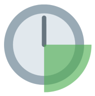

Your highest average confidence levels were from 3 PM to 6 PM during yoga weeks

MOOD

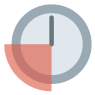

Your lowest average mood levels were from 6 PM to 9 PM during mindfulness weeks

MOOD

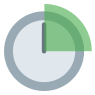

Your highest average mood levels were from 12 PM to 3 PM during mindfulness weeks

PAIN

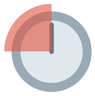

Your highest average pain levels were from 9 AM to 12 PM during brisk walk weeks

PAIN

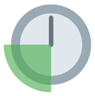

Your lowest average pain levels were from 6 PM to 9 PM during mindfulness weeks

FATIGUE

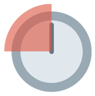

Your highest average fatigue levels were from 9 AM to 12 PM during mindfulness weeks

FATIGUE

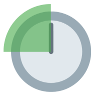

Your lowest average fatigue levels were from 9 AM to 12 PM during brisk walk weeks

Please see the "Momentary Patterns" video for an explanation of this page.

Momentary Patterns, cont'd

The symbols describe what kind of change you experienced. The ✓ shows a *positive change*, while the ✗ shows a *negative change*. A ⦸ with a line through it shows *minimal change*.

*Compared to **Baseline** weeks...*

Yoga

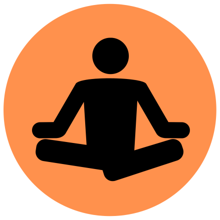

- ✗ Stress levels increased by 40%*
- ✓ Concentration levels increased by 15%*
- ✓ Confidence levels increased by 50%*
- ✗ Mood levels decreased by 11%
- ✓ Pain levels decreased by 22%*
- ✓ Fatigue levels decreased by 8%

Mindfulness Meditation

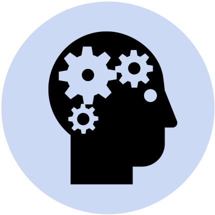

- ✗ Stress levels increased by 52%*
- ✓ Concentration levels increased by 28%*
- ✓ Confidence levels increased by 63%*
- ✗ Mood levels decreased by 10%
- ✓ Pain levels decreased by 15%*
- ✓ Fatigue levels decreased by 8%

Brisk Walk

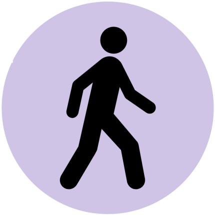

- ⦸ Stress levels had minimal change *
- ⦸ Concentration levels had minimal change
- ✗ Confidence levels decreased by 20%
- ✗ Mood levels decreased by 5%
- ✓ Pain levels decreased by 8%
- ✗ Fatigue levels increased by 15%

Please see the "Momentary Patterns" video for an explanation of this page.

An asterisk (*) next to a result means the result was statistically significant.

Adherence

Adherence is based on how closely you followed study protocol. You are adherent when you wear your Fitbit, complete your interventions, and answer your surveys. The more adherent you are, the more accurate your results are.

Total Adherence Score

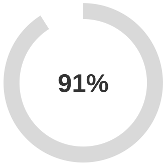

	Weeks	Fitbit							Intervention	Surveys
Yoga	10/21 − 10/27	⌚	×	⌚	⌚	⌚	⌚	⌚		Daily: 96%
	10/28 − 11/03	⌚	⌚	⌚	⌚	⌚	×	⌚	100%	Check−Ins: 92%
	12/30 − 01/05	⌚	⌚	×	⌚	⌚	⌚	⌚		Weekly: 92%
	01/06 − 01/12	⌚	⌚	⌚	×	⌚	⌚	⌚		
Mindfulness	11/04 − 11/10	⌚	⌚	⌚	⌚	⌚	⌚	×		Daily: 93%
	11/11 − 11/17	×	⌚	⌚	⌚	⌚	⌚	⌚	92%	Check−Ins: 84%
	12/16 − 12/22	⌚	⌚	⌚	⌚	⌚	⌚	⌚		Weekly: 84%
	12/23 − 12/29	⌚	⌚	⌚	⌚	⌚	⌚	⌚		
Brisk Walk	11/18 − 11/24	⌚	⌚	×	×	×	⌚	⌚		Daily: 82%
	11/25 − 12/01	⌚	⌚	⌚	⌚	⌚	⌚	⌚	96%	Check−Ins: 90%
	12/02 − 12/08	⌚	⌚	⌚	⌚	⌚	⌚	⌚		Weekly: 90%
	12/09 − 12/15	⌚	×	⌚	⌚	⌚	×	⌚		
		Total Fitbit Adherence: 87%							Total Intervention Adherence: 96%	Total Survey Adherence: 90%

Please see the "Adherence" video for an explanation of this page.

Written Summary (Other Positive Summaries)

This page summarizes information on the interventions that did not have as positive of an effect on your momentary stress. The colored boxes discuss how these interventions may have impacted your **momentary stress** as well as other health benefits.

During yoga weeks, you reported:

- This improvement was not greater than the improvements from mindfulness meditation.
- This improvement was statistically significant.

Compared to baseline, yoga also improved your:

- ✓ Steps*
- ✗ Sleep Duration
- ✗ Weekly Activity*
- ✓ Sleep Efficiency
- ✓ Average Minutes Awake
- ✓ Concentration*
- ✓ Confidence*
- ✓ Mood
- ✓ Pain*
- ✓ Fatigue

During brisk walking weeks, you reported:

- This improvement was not greater than the improvements from mindfulness meditation.
- This improvement was statistically significant.

Compared to baseline, brisk walking also improved your:

- ✗ Steps
- ✓ Sleep Duration*
- ✓ Weekly Activity*
- ✓ Sleep Efficiency
- ✓ Average Minutes Awake*
- ✗ Concentration
- ✓ Confidence
- ✗ Mood
- ✓ Pain
- ✗ Fatigue

NOTE: This report is not meant to offer medical advice. The goal of this report is to help you understand how stress interventions may impact your self−reported symptoms. You should seek a medical professional's opinion if you have any questions or concerns.

Please see the "Summary of Results" video for an explanation of this page.
An asterisk (*) next to a result means the result was statistically significant.

Personalized Trial for Stress Management

This report uses some statistical language to explain your results.

One term used throughout the report is **"statistical significance"**. **Statistical significance** suggests that there is good evidence that a treatment (for example, mindfulness meditation) is impacting the outcome (for example, stress).

When you see this symbol, the result was *statistically significant

Results that are not statistically significant are not "bad" results. Scientists are just not as confident that the results you're seeing could be related to the treatment. Similarly, if you receive statistically significant results, this does not mean that the treatment caused the result for certain. Scientists are simply *more* confident that there is a relationship between the treatment and results when there is statistical significance.

---

[1]Davidson KW, Silverstein M, Cheung K, Paluch RA, Epstein LH. Experimental Designs to Optimize Treatments for Individuals: Personalized N-of-1 Trials. JAMA Pediatr. 2021;175(4):404–409. doi:10.1001/jamapediatrics.2020.5801.

[2]Tudor-Locke, C., Craig, C.L., Brown, W.J., Clemes, S.A., De Cocker, K., Giles-Corti, B., Hatano, Y., Inoue, S., Matsudo, S.M., Mutrie, N., Oppert, J.M., Rowe, D.A., Schmidt, M.D., Schofield, G.M., Spence, J.C., Teixeira, P.J., Tully, M.A., & Blair, S.N. (2011). How many steps/day are enough? For adults. The international journal of behavioral nutrition and physical activity, 8, 79. doi:10.1186/1479-5868-8-79.
