# Supplementary material for: Protocol for randomized personalized trial for stress management compared to standard of care
Source: Front Psychol. 2023 Sep 19;14:1233884. doi: 10.3389/fpsyg.2023.1233884 (PMC10546313; doi:10.3389/fpsyg.2023.1233884)
Supplement: Supplementary file 2 [file Data_Sheet_2.PDF]

Personalized Trial for Stress Management

Your Stress Therapy Results

TestID_01

12/01/2021 - 12/08/2021

NOTE: This report is not meant to offer medical advice.The goal of this report is to help you understand how the stress interventions may impact your self−reported stress. You should seek a medical professional's opinion if you have any questions or concerns.

Feinstein Institutes for Medical Research
Northwell Health

This study was made possible by NIH (R01LM012836) and PCORI (ME-1403-12304).

Visual Summary

Our study team has determined that the most reliable measure of overall stress is momentary stress. This was measured through the three daily Ecological Momentary Assessments (the check-in surveys) you completed during the trial. This page of your report shares which month you experienced **lowest momentary stress levels**, plus other health benefits during the three months.

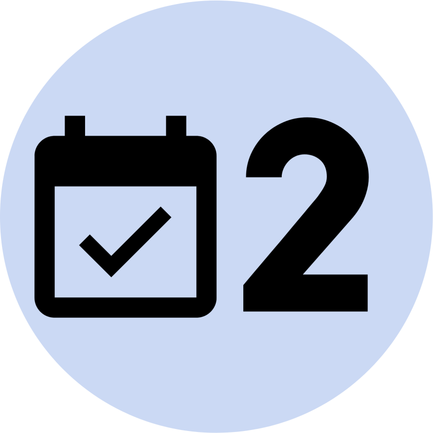

Your stress level was at its lowest during month 2!

During month 2, you reported the **lowest momentary stress** measured in the moment than months 1 or 3.

Compared to baseline, month 2 also improved your:

- ✓ Steps
- ✓ Sleep Duration
- ✓ Weekly Activity
- ✓ Sleep Efficiency
- ✓ Average Minutes Awake
- ✓ Concentration
- ✓ Confidence
- ✓ Mood
- ✓ Pain
- ✓ Fatigue

Other data presented in this report give information about other impacts of all of the months. These may be helpful in you drawing conclusions about wellbeing techniques that are beneficial **to you**. We encourage you to read the **whole report**, and to pay attention to all benefits and drawbacks presented to you.

Please see the "Visual Summary" video for an explanation of this page.

Fitbit Averages

Compared to **baseline** weeks, you had a *decrease* in sleep duration and activity and an *increase* in steps during month 1.

Compared to **baseline** weeks, you had an *increase* in steps, sleep duration, and activity during month 2.

Compared to **baseline** weeks, you had a *decrease* in steps and an *increase* in sleep duration and activity during month 3.

	Daily Steps	Nightly Sleep	Weekly Activity
Baseline	4,936 steps	306 minutes	200 minutes
Month 1	+3,000 steps	−1 minute	−110 minutes
Month 2	+3,000 steps	+3 minutes	+20 minutes
Month 3	−110 steps	+3 minutes	+55 minutes

Please see the "Fitbit Averages" video for an explanation of this page.

Daily Momentary Assessment

Momentary Stress

Each day at 3 random times you were asked to rate your stress in that moment, which all together is your overall *momentary stress*. Experiencing stress levels in the “Not at All” to “A Little Bit” range is ideal, and is reflected by the bar charts below. The donut charts above the bar charts display the total percent of your daily survey adherence.

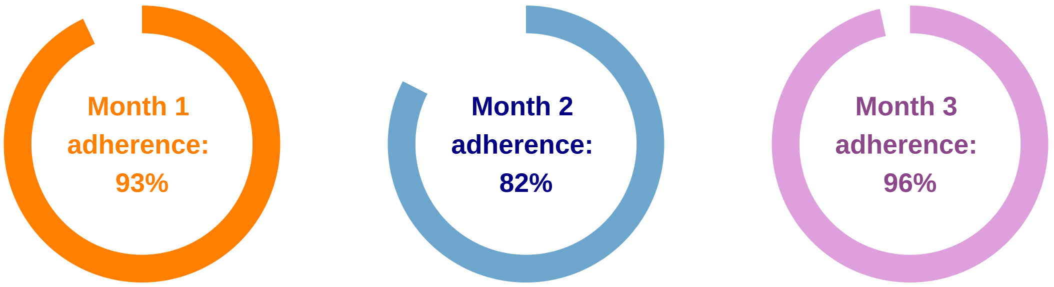

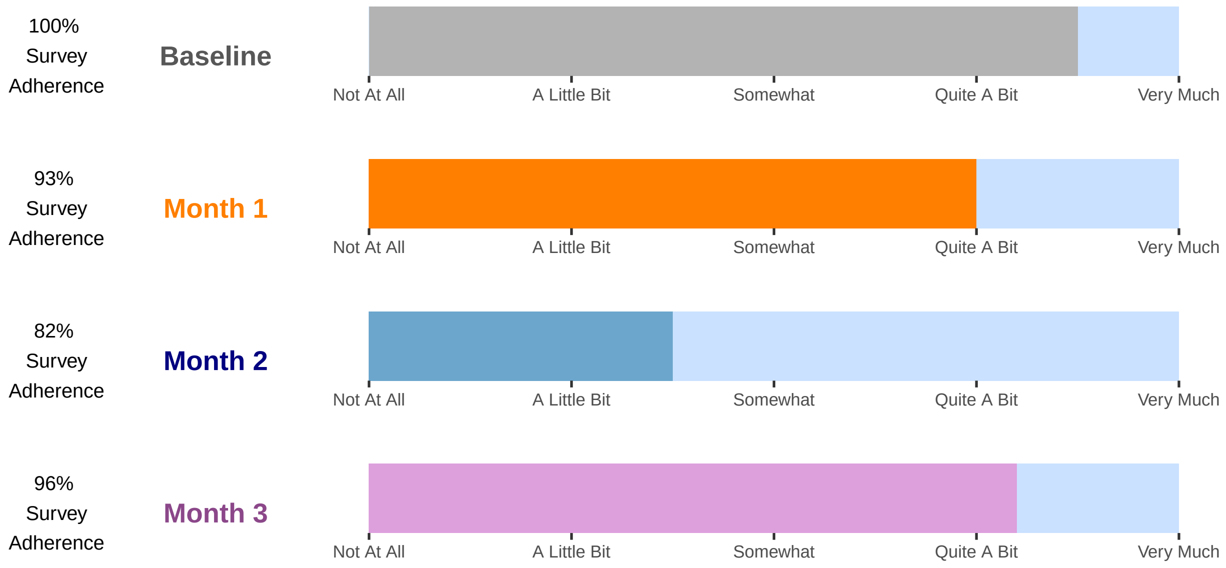

You reported *more* momentary stress during month 1 than during your **baseline**.

You reported *less* momentary stress during **month 2** than during your **baseline**.

You reported *more* momentary stress during month 3 than during your **baseline**.

Please see the "Daily Assessment" video for an explanation of this page.

Side Effects

You reported experiencing side effects 5 days of the study. The treatment that you experienced the least number of days with side effects during is ideal.

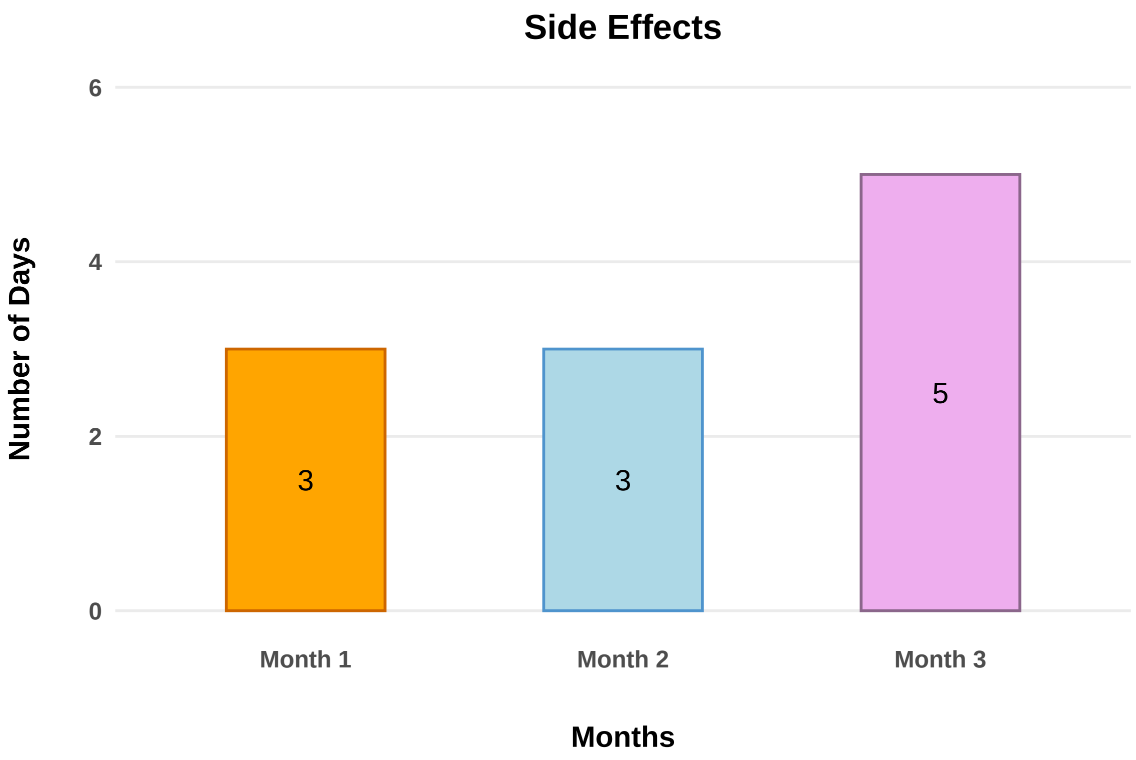

You reported the *least* number of days with side effects during **month 1** and **month 2** weeks.

Please see the "Daily Assessment" video for an explanation of this page.

Sleep Hygiene

Sleep Efficiency

Sleep Efficiency is based on the total time in bed actually spent sleeping, and is a percentage. Sleep efficiency that is greater than or equal to 85% is an indicator of good sleep quality[3].

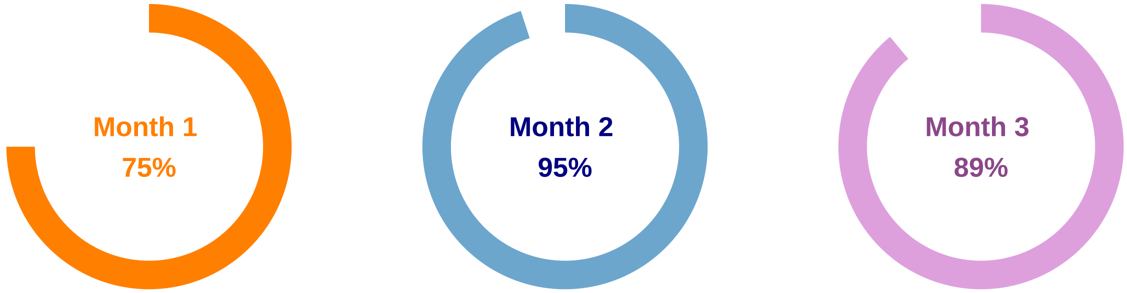

You experienced the *most* efficient sleep while completing **month 2** sessions.

Average Minutes Awake

Average minutes awake is the average number of minutes you spent awake each night after falling asleep according to your Fitbit. *Less* minutes awake throughout the night is ideal.

Month 1	Month 2	Month 3
34 minutes	18 minutes	24 minutes

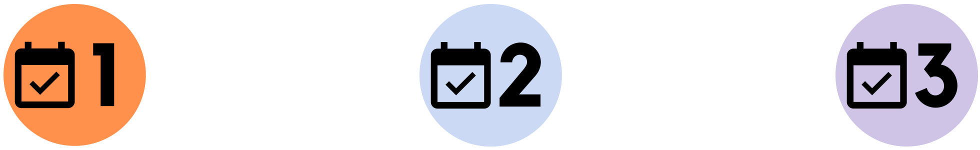

You spent the *least* average minutes awake at night while completing **month 2** sessions.

Please see the "Sleep Hygiene" video for an explanation of this page.

Momentary Patterns

Every day at three random times you were asked to rate your momentary state in that moment, which all together are your *momentary patterns*.

STRESS

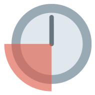

Your highest average stress levels were from 6 PM to 9 PM during month 2

STRESS

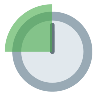

Your lowest average stress levels were from 9 AM to 12 PM during month 1

CONCENTRATION

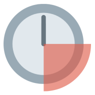

Your lowest average concentration levels were from 3 PM to 6 PM during month 3

CONCENTRATION

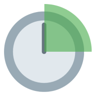

Your highest average concentration levels were from 12 PM to 3 PM during month 3

CONFIDENCE

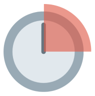

Your lowest average confidence levels were from 12 PM to 3 PM during month 1

CONFIDENCE

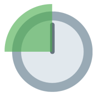

Your highest average confidence levels were from 9 AM to 12 PM during month 1

MOOD

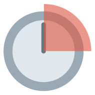

Your lowest average mood levels were from 12 PM to 3 PM during month 3

MOOD

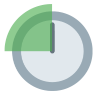

Your highest average mood levels were from 9 AM to 12 PM during month 1

PAIN

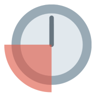

Your highest average pain levels were from 6 PM to 9 PM during month 1

PAIN

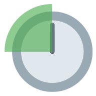

Your lowest average pain levels were from 9 AM to 12 PM during month 3

FATIGUE

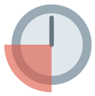

Your highest average fatigue levels were from 6 PM to 9 PM during month 2

FATIGUE

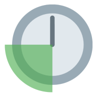

Your lowest average fatigue levels were from 6 PM to 9 PM during month 3

Please see the "Momentary Patterns" video for an explanation of this page.

Momentary Patterns, cont'd

The symbols describe what kind of change you experienced. The ✓ shows a *positive change*, while the ✗ shows a *negative change*. A ⃠ with a line through it shows *minimal change*.

*Compared to **Baseline** weeks...*

Month 1

- ✗ Stress levels increased by 40%
- ✓ Concentration levels increased by 15%
- ✓ Confidence levels increased by 50%
- ✗ Mood levels decreased by 11%
- ✓ Pain levels decreased by 22%
- ✓ Fatigue levels decreased by 8%

Month 2

- ✗ Stress levels increased by 52%
- ✓ Concentration levels increased by 28%
- ✓ Confidence levels increased by 63%
- ✗ Mood levels decreased by 10%
- ✓ Pain levels decreased by 15%
- ✓ Fatigue levels decreased by 8%

Month 3

- ⃠ Stress levels had minimal change
- ⃠ Concentration levels had minimal change
- ✗ Confidence levels decreased by 20%
- ✗ Mood levels decreased by 5%
- ✓ Pain levels decreased by 8%
- ✗ Fatigue levels increased by 15%

Please see the "Momentary Patterns" video for an explanation of this page.

Adherence

Adherence is based on how closely you followed study protocol. You are adherent when you wear your Fitbit, view the videos, and answer your surveys. The more adherent you are, the more accurate your results are.

Total Adherence Score

83%

	Weeks	Fitbit	Videos	Surveys
Month 1	10/21 – 10/27	⌚ ✗ ⌚ ⌚ ⌚ ⌚ ⌚	22%	Daily: 93%
	10/28 – 11/03	⌚ ⌚ ⌚ ⌚ ⌚ ✗ ⌚		Check−Ins: 84%
	11/04 – 11/10	⌚ ⌚ ⌚ ⌚ ⌚ ⌚ ✗		Weekly: 84%
	11/11 – 11/17	✗ ⌚ ⌚ ⌚ ⌚ ⌚ ⌚		
Month 2	11/18 – 11/24	⌚ ⌚ ✗ ✗ ✗ ⌚ ⌚	14%	Daily: 82%
	11/25 – 12/01	⌚ ⌚ ⌚ ⌚ ⌚ ⌚ ⌚		Check−Ins: 90%
	12/02 – 12/08	⌚ ⌚ ⌚ ⌚ ⌚ ⌚ ⌚		Weekly: 90%
	12/09 – 12/15	⌚ ✗ ⌚ ⌚ ⌚ ✗ ⌚		
Month 3	12/16 – 12/22	⌚ ⌚ ⌚ ⌚ ⌚ ⌚ ⌚	8%	Daily: 96%
	12/23 – 12/29	⌚ ⌚ ⌚ ⌚ ⌚ ⌚ ⌚		Check−Ins: 92%
	12/30 – 01/05	⌚ ⌚ ✗ ⌚ ⌚ ⌚ ⌚		Weekly: 92%
	01/06 – 01/12	⌚ ⌚ ⌚ ✗ ⌚ ⌚ ⌚		
		Total Fitbit Adherence: 87%	Total Video Adherence: 53%	Total Survey Adherence: 90%

Please see the "Adherence" video for an explanation of this page.

Written Summary (Other Positive Summaries)

This page summarizes information on the interventions that did not have as positive of an effect on your momentary stress. The colored boxes discuss your **momentary stress** as well as other health benefits were impacted during these months.

During month 1, you reported that your momentary stress was higher than that of month 2.

Compared to baseline, month 1 also improved your:

- ✓ Steps
- ✗ Sleep Duration
- ✗ Weekly Activity
- ✓ Sleep Efficiency
- ✓ Average Minutes Awake
- ✓ Concentration
- ✓ Confidence
- ✓ Mood
- ✓ Pain
- ✓ Fatigue

During month 3, you reported that your momentary stress was higher than that of month 2.

Compared to baseline, month 3 also improved your:

- ✗ Steps
- ✓ Sleep Duration
- ✓ Weekly Activity
- ✓ Sleep Efficiency
- ✓ Average Minutes Awake
- ✗ Concentration
- ✓ Confidence
- ✗ Mood
- ✓ Pain
- ✗ Fatigue

NOTE: This report is not meant to offer medical advice. The goal of this report is to help you understand how stress interventions may impact your self−reported symptoms. You should seek a medical professional's opinion if you have any questions or concerns.

Please see the "Summary of Results" video for an explanation of this page.

Personalized Trial for Stress Management

This report does not use statistical language to explain your results.

As part of the standard care arm, your report was not analyzed for **"statistical significance"**. **Statistical significance** suggests that there is good evidence that a treatment (for example, mindfulness meditation) is impacting the outcome (for example, stress).

When you see this symbol, the result was *statistically significant

Results that are not statistically significant are not "bad" results. Scientists are just not as confident that the results you're seeing could be related to the treatment. Similarly, if you receive statistically significant results, this does not mean that the treatment caused the result for certain. Scientists are simply *more* confident that there is a relationship between the treatment and results when there is statistical significance.

---

[1]Davidson KW, Silverstein M, Cheung K, Paluch RA, Epstein LH. Experimental Designs to Optimize Treatments for Individuals: Personalized N-of-1 Trials. JAMA Pediatr. 2021;175(4):404–409. doi:10.1001/jamapediatrics.2020.5801.

[2]Tudor-Locke, C., Craig, C.L., Brown, W.J., Clemes, S.A., De Cocker, K., Giles-Corti, B., Hatano, Y., Inoue, S., Matsudo, S.M., Mutrie, N., Oppert, J.M., Rowe, D.A., Schmidt, M.D., Schofield, G.M., Spence, J.C., Teixeira, P.J., Tully, M.A., & Blair, S.N. (2011). How many steps/day are enough? For adults. The international journal of behavioral nutrition and physical activity, 8, 79. doi:10.1186/1479-5868-8-79.
