# Supplementary material for: Protocol for randomized personalized trial for stress management compared to standard of care
Source: Front Psychol. 2023 Sep 19;14:1233884. doi: 10.3389/fpsyg.2023.1233884 (PMC10546313; doi:10.3389/fpsyg.2023.1233884)
Supplement: Supplementary file 3 [file Table_1.docx]

Appendix A: Informed Consent Form

**Northwell Health**

**Consent for Participation in a Research Study**

**Study Title:** Personalized Trials for Stress Management Against Standard of Care

**Principal Investigator:** Karina W. Davidson, PhD, MASc

**Sponsor:** National Institutes of Health (NIH)

**Protocol Number: 21-0968-MRB**

**Consent Version Date: 10/18/2022**

**About this research**

You are being asked to participate in a research study.

This consent form will give you information about the study to help you decide whether you want to participate. Please read this form, and ask any questions you have, before agreeing to be in the study.

**Important Information**

This information gives you an overview of the research. More information about these topics may be found in the pages that follow.

| **Why am I being asked to provide my consent?** | This is a research study, which is different than personal medical care.  Scientists do research to answer important questions which might help change or improve the way we do things in the future. |
| --- | --- |
| **Do I have to join this research study?** | No. Taking part in this research study is voluntary.  You may choose not to take part in this study or may choose to leave the study at any time.  Deciding not to participate, or deciding to leave the study later, will not result in any penalty or loss of benefits to which you are entitled.  This study may enroll employees of Northwell Health. Employee participation or non-participation will have no bearing on an employee’s position at Northwell Health.  This study may also enroll students, including those that may attend Hofstra University. Student participation or non-participation will have no bearing on a student’s grades or academic standing. |
| \| **Why is this research study being done?** \|  \| \| --- \| --- \| | The main purpose of this study is to determine if an N-of-1 study design, or what we are calling “Personalized Trials”, can improve health outcomes over common stress management techniques such as mindfulness meditation, yoga, or brisk walking or other stress management practices that may be used without any prompts from the study team (standard practice) . |
| **What will happen to me during the study?** | In this study, you will test the effects of three different stress management interventions to improve your reported stress. You will be randomly assigned to one of three groups or “arms” and will test out guided mindfulness meditation, guided yoga, and guided brisk walking interventions delivered via an online pre-recorded video. Everyone will receive a personalized report after they have tested each intervention. The report will tell participants information about their personal response to each intervention. Everyone will then be able to select which intervention they liked best, so they can receive an additional two weeks access to that specific video. At the end of the study, all participants will be sent a satisfaction survey. Some participants will be randomly selected and invited to participate in an audio-recorded follow up interview to discuss their experiences at study end. For a detailed description of study procedures, see the below section “What will happen in this research study.” |
| **How long will I participate?** | This study will take place over the course of 18 weeks. There is a 2-week baseline period to assess if you are eligible to proceed to the intervention period, a 12-week intervention period, a 2-week observation period with no interventions so that we can generate your own personalized report, and an additional 2 –week period of completing your selected intervention. At the end of the 18-week study period, you will be asked to complete a satisfaction survey and some participants will be randomly invited to participate in a follow-up video conference or phone call to talk about their experience. |
| **Will taking part expose me to risks?** | This research presents minimal risk to you. Some risks that you may experience could be: mild side effects from yoga and/or brisk walking like fatigue or sore muscles, a loss of confidentiality or privacy, like having your personal information shared with someone who is not on the study team and was not supposed to see or know about your information, and possibly mild skin irritation from wearing your Fitbit activity tracker. You may also experience unpleasant feelings as you are answering some of the survey questions about your mood, or you could also experience unpleasant feelings during your mindfulness meditation sessions. |
| **Are there any benefits to participation?** | This study may or may not benefit you. Sometimes yoga and brisk walking increase your chance of being healthier, and mindfulness meditation can sometimes result in pleasant experiences. Although you may not experience any personal, physical benefit from participation, there is a chance that participation in this study may help you to better understand your individual response to different stress interventions so that you can learn how to manage stress. |
| **What are my alternatives to participation?** | You do not need to participate in this study to receive common stress management interventions. There are many programs that can be followed for stress management. Please consult with your care provider for additional alternatives. |

**Please review the rest of this document for details about these topics and additional things you should know before making a decision about whether to participate in this research.**

**Introduction and Research Purpose:**

You are being asked to join a research study. The main purpose of this study is to determine if an N-of-1 study design, or what we are calling “Personalized Trials” can improve health outcomes over common stress management techniques such as mindfulness meditation, yoga, or brisk walking or other stress management practices that may be used without any prompts from the study team (standard practice).

If you are a patient at Northwell Health, you do not have to be in this study to receive medical care. You should ask questions before you decide if you want to participate. You can also ask questions at any time during the study by emailing [personalizedtrials@northwell.edu](mailto:personalizedtrials@northwell.edu) or calling (646) 995-8958.

**Why is this research study being done?**

This particular study uses Personalized Trials to help people evaluate the effects of three different stress management interventions to improve individual self-report of perceived stress: guided mindfulness meditation, guided yoga, and guided brisk walking. You are being asked to participate in this study because you have reported perceived stress.

If you will be traveling outside of the United States during the study period, or if you will not have access to text-messaging or internet for more than a few days during the study period, you should talk to a member of the study team to determine your eligibility for this study.

**How many people will take part in this study?**

This research study hopes to enroll up to 1000 people. We hope to randomize 212 people to participate in the stress management interventions (53 in Arm 1, 53 in Arm 2 and 106 in Arm 3).

**How long will you be in this study?**

If you are selected to take part, you will participate in this study for 18 weeks and involves the following phases:

- Baseline (2 weeks)
- Intervention (12 weeks)
- Report generation (2 weeks)
- Observation (2 weeks)

Some people will be selected to have a phone call or a video conference after the 18 weeks described above. The call will last about 60 minutes.

**What will happen in this research study?**

After filling out this consent form, you will be asked to provide the research team with some more information about yourself. This will include your home address, the times you wake up each morning and go to sleep at night, and preferred time to receive text messages and surveys for the study. ***This study uses text-message reminders to help you through the study protocol. Completing the surveys will require cellular data if you are not connected to Wi-Fi. You will not be reimbursed for text messages or data charges, and standard carrier rates may apply.***

**Baseline**

You will be sent a secure text message confirming the start date of your baseline period. This secure message will include a short video that explains the study again, and also what to expect during this baseline period. If you do not have your own Fitbit device, or one that is acceptable for this study, you will be sent a Fitbit device prior to the start of your baseline period. If you do not already have the Fitbit app downloaded on your phone, you will be asked to do so in order to participate in this research. If you have already downloaded the Fitbit app on your phone, you will be sent instructions to log out of your personal Fitbit account, and log in to a study account using a provided study ID. During baseline you will not receive any stress interventions; ***please continue to manage your stress as you normally would.*** Each evening you will receive a text message survey asking them to report if you did anything to relieve your stress that day, and report any experienced side effects you attribute to that activity. You will also receive three text messages each day at random times (like the flip of a coin) asking you to rate your pain, fatigue, concentration, confidence, mood, and stress levels at that exact moment.  You will be asked to wear your Fitbit device all day and night, even while you are sleeping. You will receive text messages to sync your Fitbit device by opening the Fitbit app on their phone, and reminders to charge your device if it has a low battery. On Sundays, you will be asked to complete a slightly longer stress-related survey. If you are at least 80% adherent to text message surveys and Fitbit wear you will be eligible for participation in the intervention phase.

**Intervention**

If there is enough data collected during your baseline period, and you are eligible to participate in this phase, you will be randomly assigned (like the flip of a coin) to one of three research groups or “arms.”

In Arms 1 and 2, participants will receive a Personalized Trial of the 3 stress management techniques (guided mindfulness meditation, yoga, and brisk walking). Participants will be assigned to a sequence order of either ABCCBA or CBAABC, where each letter represents two weeks of focused intervention (A= mindfulness meditation, B= yoga, and C= brisk walking). For instance, in the ABCCBA group, participants would be asked to complete mindfulness meditation for two weeks, yoga for two weeks, brisk walking for 4 weeks, yoga again for 2 weeks and end the intervention period with mindfulness meditation for 2 weeks. Participants in both sequences will be prompted to complete 30-minute stress management sessions during the applicable intervention weeks and will be limited to three views of study-provided stress management content/videos each week. Participants will be asked to report when they viewed this content, as well as additional stress management techniques completed, in a daily survey.

- In Arm 3, participants will receive access to the same stress management techniques (guided mindfulness meditation, guided yoga, and guided walking), including the same number of views of the content as participants in Arms 1 and 2, ***but participants will not be prompted via text message to complete any of the sessions a specific number of times in a given week***. Participants in Arm #3 will be re-sent the link to access their stress management content/videos on a weekly basis, and will be limited to 36 total views of study-provided stress management content. Participants will be asked to report when they viewed this content, as well as additional stress management techniques completed, in a daily survey.

During the intervention period, you will continue wearing their Fitbit device each day and night, and continue to answer the same surveys from the baseline period. You will continue to be asked to report any intervention-related side effects you may be experiencing via your daily survey. If you are concerned about any side effects during the study, you should stop the intervention and contact a member of the research team for more information about continuing or stopping study participation.

During the study period, you may also receive additional text messages to remind you of important next steps for this study (like a reminder that you’re transitioning to a new intervention period)., We will send a maximum of 7 text messages per day during the study, unless there are issues with your data (battery, sync, etc).

**Report Generation:**

After 12 weeks of having the opportunity to view guided mindfulness meditation, yoga, and brisk walking content for stress management, you will continue to answer daily surveys and wear your Fitbit device for 2 weeks. However, you will not receive any additional stress management content during this time to allow for a personalized report of your observed data to be generated by the study team.

**Observation:**

Once the personalized report has been generated and provided to you, you will be asked to select ***one stress management technique*** that you would like to continue with for the observation period, which includes another 6 sessions (2 weeks) of stress management content (e.g. if yoga is selected, you will receive 6 additional views of the yoga content over the course of 2 weeks).   At the end of the 2-week observation period, all participants will be asked to complete a study satisfaction survey that asks about your opinion on Personalized Trials. This survey will be delivered by secure messaging, phone or video conference.

This is a picture of what participation in this research involves:


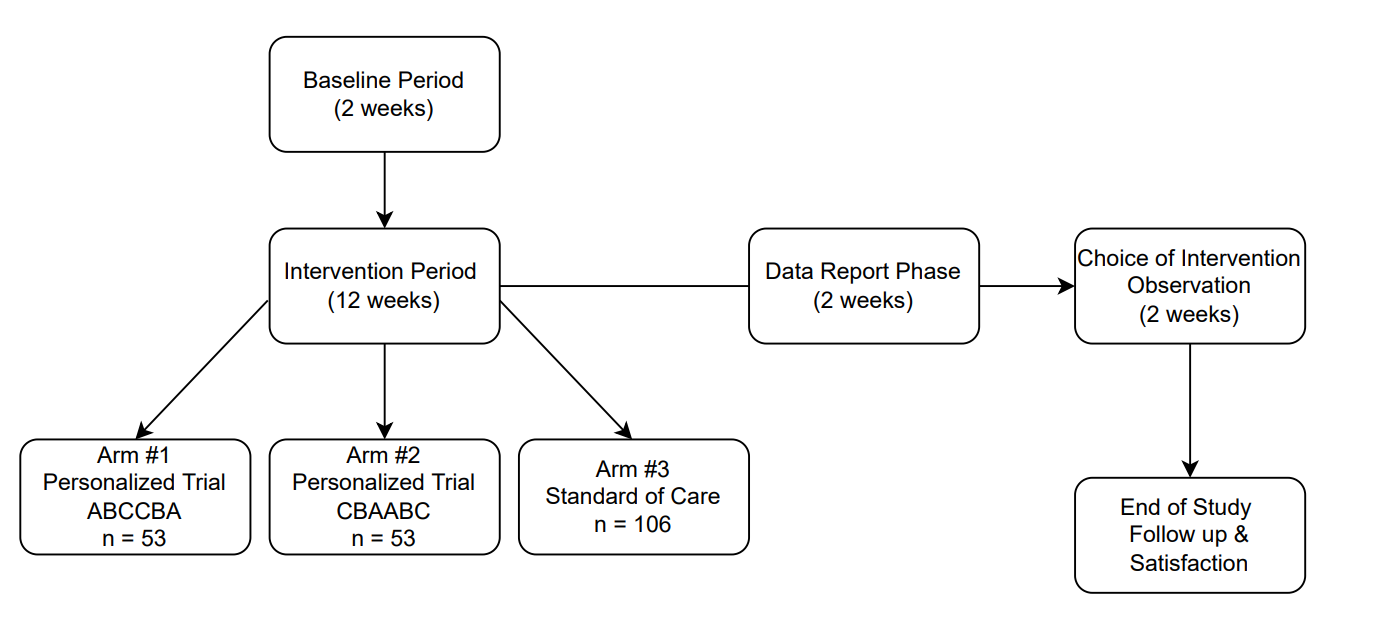


We will also invite some participants from each group or “arm” to schedule a video conference or call to discuss their experience. We will also ask about your opinion on Personalized Trials. This interview will be audio-recorded and transcribed for data collection purposes only; however, if you decline to be audio recorded, you may still participate in the follow-up interview.

Participation in this study is over after you have completed 18 weeks of data collection and your satisfaction survey. If you are selected to participate in an interview, your participation will end after completion of the interview.

**What are the risks of the research study? What could go wrong?**

This study is considered minimal risk which means that the risks of participating in this study are not greater than risks you may encounter in your daily life or during routine physical or psychological examinations or tests. This section explains the potential risks of participating in this research.

Fitbit Band:

You may experience mild skin irritation (rash) from wearing the Fitbit band during this research study.  To reduce irritation, keep the band clean and dry. To provide relief for you skin if this mild risk occurs, remove the band for a short period of time.

Intervention Side Effects:

You may feel fatigued or experience muscle soreness from increased low-intensity walking or yoga. Mindfulness meditation may cause experiences that are pleasant, unpleasant, or neutral. It can lead to states of ease, joy, relaxation, peace and a sense of wellbeing. Unpleasant experiences such as agitation, physical discomfort, sleepiness, sadness and anger are also common. Such experiences are usually temporary.  If you are concerned about any of these side effects or any others you may experience, you should immediately stop the intervention and contact a member of the study team. You may still be able to continue with the study or you may be asked to end your participation in the study.

Loss of Confidentiality or Privacy:

There is the possibility of a loss of confidentiality or privacy. Loss of privacy means having your personal information shared with someone who is not on the study team and was not supposed to see or know about your information. The study team plans to protect your privacy by only sharing necessary information about you to those outlined in the “Who else will see your information?” section below.

Survey:

Some of the questions we will ask you are personal. You may feel embarrassed or stressed. You may ask to see the questions before deciding whether or not to take part in this study.

**What are the benefits of this research study?**

This study may or may not benefit you. Sometimes yoga and brisk walking increase your chance of being healthier, and mindfulness meditation can sometimes result in pleasant experiences. Although you may not experience any personal, physical benefit from participation, there is a chance that participation in this study may help you to better understand your individual response to different stress interventions so that you can learn how to manage stress.

Through pooling N-of-1 trial data, a greater understanding of the effectiveness of mindfulness meditation, yoga, and brisk walking will arise. Additionally, the information collected from your involvement as a research participant will inform the development of Personalized Trials to help patients in the future to help other research participants and eventually patients discover which treatment options are best for them as an individual.

**Will I receive my results?**
**Yes, the data we collect from you during study activities could impact how you manage your stress. For this reason, much of the information collected about you during the study will be analyzed and provided back to you. You will receive this data in the form of a personalized stress management participant report. This report is meant to summarize your observed data and self-reported symptoms and should not be considered medical advice. You may wish to meet with professionals with expertise to help you learn more about your research results. The study team/study will not cover the costs of any follow-up consultations or actions.**

**If you do not want to take part in this research study, what are your other choices?**

You do not need to participate in this study in order to participate in guided yoga, mindfulness meditation or brisk walking as possible interventions for stress. There are many programs that can be followed for stress management. Please consult with your care provider for additional alternatives.

If you do not join this study, you have other choices for treatment of stress. Talk to your doctor about your choices. Your other choices may include:

- Relaxation techniques
- Lifestyle changes
- Stimulus control
- Cognitive Behavioral Therapy (CBT)
- Interventions provided in this study

**Are there any costs for being in this research study?**

This research study is funded by the National Institutes for Health (NIH). All study related equipment, devices (if applicable), and procedures will be provided to you at no cost except as noted below. Neither you nor your insurance company will be billed for your participation in this research.

This study uses text messages to deliver notifications, reminders, and study questionnaires. Standard message and data rates from your wireless carrier may apply. You will not be compensated for any costs related to data usage or sending or receiving text messages by the study or by members of the study team.

**Will you receive any payments for participating in this research study?**

All participants who are in their intervention weeks (weeks 2-14) are eligible to receive a $100 Clincard. Each week, a participant’s name will be randomly selected and if the person selected has maintained at least 80% adherence to survey responses and Fitbit wear for that week, they will be given a $100 Clincard via mail. If the selected participant has not maintained at least 80% adherence, they will be advised to try again the following week, and no other participant will be given a $100 Clincard that week. Although not likely, a participant can potentially be selected and given a $100 Clincard by random lottery up to 12 times, totaling $1200, during the study. You are responsible for paying state, federal, or other taxes for the payments you receive for being in research, if the total compensation you receive from participation in Northwell research totals $600 or more. Taxes are not withheld from your compensation.

Participants who are selected and complete the qualitative interview at the end of their study will be given a $25 Clincard via mail.

If you are a participant who was given a Fitbit device to participate in this research, you will be allowed to keep the device.

**What are your rights as a research participant?**
**Your participation in this project is voluntary.**

**This study may enroll employees of Northwell Health. Employee participation or non-participation will have no bearing on your position at Northwell Health. This study may also enroll students, including those that attend Hofstra University. Student participation or non-participation will have no bearing on your grades or standing at your academic institution.**

If you are also a Northwell Health patient, the quality of your medical care will be the same, whether you join, refuse to join, or decide to leave the study. If you do not join the study you will not be penalized or lose benefits to which you are entitled. If you join the study you may withdraw at any time without prejudice to your future care at Northwell Health.

**Could you be taken off the study before it is over?**

It is possible that your participation in this study may end without your consent. This decision may be made by a researcher, study sponsor or the Institutional Review Board (IRB- the committee that oversees research at this institution).

Reasons for withdrawal may include:

- Failure to follow the study protocol,
- Self-reported adverse side effects to two or more treatments
- It is not in your best interest to continue on this study, or
- The study is stopped.

If you are concerned about any side effects during the study, you should stop the intervention and contact a member of the research team for more information about continuing or stopping your study participation.

If you withdraw from this study or if you are withdrawn from the study, any data already collected will continue to be used.  However, no new data will be collected.  

What happens if new information is learned?

You will be told of any new findings that may change your decision to continue to participate. Your consent to continue to take part in this study may be obtained again.

**What information will be collected and used for this study?**

If you agree to be in this study, we will collect information that identifies you. We may collect the results of questionnaires, interviews, Fitbit activity and sleep, and video views. We will only collect information that is needed for the research. This information has been described in this consent form. If you sign this consent form, you are giving us permission to collect, use and share your health information. This permission is called authorization. If you do not want to provide authorization, then you cannot participate in this research study.

Data collected by Fitbit includes activity data (e.g. daily steps, heart rate, floors climbed, activity intensity),  sleep data (total sleep minutes, sleep stage estimates, sleep and wake times), and device data (last sync date and Fitbit battery level). We will only collect this data for the duration of your participation in this research. General email addresses that have been created for this research study have been used to create these study accounts without any information that could identify you. It is important that you use the Fitbit account provided during the study and NOT your personal Fitbit account (if you have one) to protect your information, and to allow us to collect your data. The research team will not share any of your personal information with Fitbit.  Once your study period is complete, you will be sent instructions on how to un-link your Fitbit device from the account.  Your IP address may be collected by the Fitbit app which could be considered identifiable information. Your Fitbit data will be securely stored in Fitbit electronic platforms as is standard for all Fitbit users. Fitbit’s Terms of Service and Privacy Policy are separate from this research consent form, but should be considered before you sign this consent form. To join this study, a Fitbit account will be created for you. Study staff will agree to the [Fitbit Terms of Service](https://www.fitbit.com/global/us/legal/terms-of-service) <https://www.fitbit.com/global/us/legal/terms-of-service>(https://www.fitbit.com/global/us/legal/terms-of-service) and [Privacy Policy](https://www.fitbit.com/global/us/legal/privacy-policy) (<https://www.fitbit.com/global/us/legal/privacy-policy>) when they create your account using a study specific username and password.  Fitbit’s Terms of Service includes information about your legal rights when using Fitbit’s products that may differ from your rights as a participant in this study.  Fitbit’s Privacy Policy describes how Fitbit collects, uses, shares, and protects your data. You can exercise your right to access your personal information by logging into your Fitbit account and using your account settings. Fitbit may also have access to device identifiers so they may be able to identify that you are a participant in this research. For more information about the information that Fitbit may have access to, refer to Fitbit’s Privacy Policy.

Survey data will be collected via a secure web browser and stored in HIPAA-compliant, Northwell approved platforms: N1Thrive and REDCap. Screening and consent procedures will be delivered by REDCap. N1Thrive is a company that the study team is partnering with to develop the technology to analyze data for current and future Personalized Trials. N1Thrive will send study messages and links to the intervention and surveys on behalf of the study team. Text messages will alert you to a new message from “N1Thrive” and contain a link to open this secure browser directly on your phone. No identifying information will be shared via text message.

Data from your Fitbit device may be shared with Northwell Health for research purposes through an online portal called Fitabase. The study account given to you to connect your Fitbit will be linked to an identification number in the Fitabase system. No information that could be used to identify you will ever be shared with Fitabase. Only the research team will have access to data that will be able to connect a research participant to their Fitabase ID. Fitabase will stop sharing your data at the end of your study, but as an added step, you will be asked to remove the study account from your device if you would like to keep your Fitbit.

The study team will have direct access to the survey and Fitbit data shared through N1Thrive. We will do everything we can to keep your data secure, however, complete confidentiality cannot be promised.

We will also collect your email address and phone number, mailing address and demographic information. Any information collected during this study that can identify you by name will be kept confidential. We will only collect information that is needed for the research. This information has been described in this consent form. If you sign this consent form, you are giving us permission to collect, use and share your health information. This permission is called authorization. If you do not want to provide authorization, then you cannot participate in this research study.

**Who else will see your information?**

Study records that identify you will be kept private. You will not be identified in study records or publications disclosed outside Northwell Health, except as detailed below.

Investigators will share information collected from this research study with:

- Study sponsor (NIH) and/or its agents,
- Other researchers,
- Accrediting agencies,
- Data safety monitoring board.

The following reviewers may access your study records to make sure that this study is being done properly:

- Representatives from Federal and state government oversight agencies, such as the Department of Health and Human Services, and the National Institutes of Health,
- Representatives from Northwell Health’s Human Research Protection Program (a group of people that oversee research at this institution)

We will do our best to protect the privacy of your records but it is possible that once information is shared with people listed on this form, it may be released to others. If this happens, your information may no longer be protected by the federal law. In the future, we may publish results of this study in scientific journals and may present it at scientific meetings. If we do, we will not identify you.  If the researchers learn about potential serious harm to you or someone else or other public health concerns, it will be shared with the appropriate authorities.

**How long will your health information be kept?**
**There is no limit on the length of time we will keep your** information for this research because it may be analyzed for many years. We will keep it as long as it is useful, unless you decide you no longer want to take part or we close the study. You are allowing access to this information indefinitely.

**Can you change your mind?**

If you change your mind about being in the study, you may withdraw at any time. If you want us to stop collecting your health information, you need to send an email to the research team at the following address: [personalizedtrials@northwell.edu,](mailto:personalizedtrials@northwell.edu) or via secure message on n1thrive.

Your text or email needs to say that you have changed your mind and do not want the researcher to collect and share your health information. You may also need to leave the research study if we cannot collect any more health information. We may still use the information we have already collected. We need to know what happens to everyone who starts a research study, not just those people who stay in it.

**Will information about this study be available to the public?**

The researcher also plans to share information about the study, including de-identified data, on the following data sharing website: <https://cos.io/>. The Open Science Framework is a free, open-source web application built to provide researchers with a free platform for data and materials sharing. There will be no identifiable data posted to this website or used in future studies. In addition, a description of this clinical trial will be available on [http://www.Clinical Trials.gov](http://www.feinsteininstitute.org/Feinstein/Clinical+Trials) , as required by U.S. Law. This Web site will not include information that can identify you. At most, the Web site will include a summary of the results. You can search this website at any time.

**Certificate of Confidentiality**

To help us protect your privacy, this research is covered by a Certificate of Confidentiality from the US Department of Health and Human Services (DHHS). The Certificate of Confidentiality means that researchers cannot be forced to identify you, even under a court subpoena. The Certificate does not mean the Secretary of DHHS approves or disapproves of the project. It adds special protection for the research information about you. You should know, however, that researchers may provide information to appropriate individuals or agencies if harm to you, harm to others or child abuse becomes a concern. A Certificate of Confidentiality does not prevent you or a member of your family from voluntarily releasing information about yourself or your involvement in this research. However, if an insurer, employer or other person learns about your participation and gets your consent to receive research information, then the researchers will have to provide your information.

**Will my information be used for research in the future?**
Information collected from you for this research may be used for future research studies, pooled with participant data from other trials conducted by the Center for Personalized Health, or shared with other researchers for future research. If this happens, information which could identify you will be removed before any information is shared. Since identifying information will be removed, there will not be an additional consent for future research. By consenting to participate in this study you are agreeing to allow your coded data to be used by future researchers without additional consent. We will also contact you and tell you about future research studies that may require your consent.

Some information collected during this study that can identify you will be kept on file. This information may be used in the future to contact you for future participation in Personalized Trials, if you’ve indicated you may be interested at the end of the study. This information will be stored on a secure database. It will only be accessible by trained members of the study team. If you change your mind about being contacted in the future, you may follow the procedures outlined above to notify the researcher.

**Does the investigator of this study receive money if you take part?**

The investigators on this study receive money to conduct the study, but do not financially benefit from your participation. The money they receive is to pay them back for the costs of conducting the research study. Funding for this research study is provided by the National Institutes of Health (NIH)*.*

**Who can answer your questions about this study?**

If you have any questions about the study, you may call Danielle Miller, Supervisor of Clinical Research, 646-995-8958 or email [personalizedtrials@northwell.edu](mailto:personalizedtrials@northwell.edu). If you have questions about side effects or injury caused by research you should call Joan Duer-Hefele RN, MA, CCRC, Director of Clinical Research, at (646) 766-7153. If you need emergency care, dial 911 or go to the nearest Emergency Room. If you have questions about your rights as a research participant, concerns about being in the study, or would like to offer input, you may contact the Office of the Institutional Review Board (the committee that oversees research at this institution) at (516) 465-1910.

A signed copy of this consent form will be made available to you.

[Signature Page Follows]Please respond to the following questions to demonstrate your understanding of study procedures and your rights as a research participant.

1. As a participant, I will need to wear my Fitbit 24 hours a day, even while I am sleeping.
   - True
   - False
2. As a participant, I will be placed in one of three groups. All groups will receive access to stress management interventions. One group will only receive a link to their video intervention but will not be prompted or reminded to complete the stress management intervention according to a schedule. The other groups will complete the interventions in different orders, and will receive prompts and reminders about completing the interventions.
   - True
   - False
3. As a participant, I can remove myself from the study at any time by contacting the research team.
   - True
   - False
4. As a participant, I will receive at most 7 text messages a day, unless there is an unexpected problem with my data.
   - True
   - False

**Summation/Signature**

You have read the above description of the research study. You have been told of the risks and benefits involved. You have also been given the opportunity to have any questions you may have answered to your satisfaction by calling 646-995-8958 or by emailing [PersonalizedTrials@northwell.edu](mailto:PersonalizedTrials@northwell.edu). A member of the research team will answer any future questions you may have. You voluntarily agree to join this study and know that you can withdraw from the study at any time without penalty. By electronically signing this form, you have not given up any of your legal rights.

____________________________________________________________

Typed Name of Participant

___________________________________________________ ____________________

Electronic Signature Date & Time Stamp

Appendix B(1): Example Participant Report [Personalized Trial, Arms 1 and 2]

Appendix B(2): Example Participant Report [Standard Care, Arm 3]

Appendix C: Ecological Momentary Assessment (EMA) for stress

Please answer the following questions according to how you're feeling at this time:

I feel stressed

0 = Not at all

1 = A little bit

2 = A little bit

3 = A little bit

4 = Somewhat

5 = Somewhat

6 = Somewhat

7 = Quite a bit

8 = Quite a bit

9 = Quite a bit

10 = Very much

Appendix D: Perceived Stress Scale (PSS-10)

In the last month, how often have you been upset because of something that happened unexpectedly?

Never

Almost Never

Sometimes

Fairly Often

Very Often

In the last month, how often have you felt that you were unable to control the important things in your life?

Never

Almost Never

Sometimes

Fairly Often

Very Often

In the last month, how often have you felt nervous and “stressed”?

Never

Almost Never

Sometimes

Fairly Often

Very Often

In the last month, how often have you felt confident about your ability to handle your personal problems?

Never

Almost Never

Sometimes

Fairly Often

Very Often

In the last month, how often have you felt that things were going your way?

Never

Almost Never

Sometimes

Fairly Often

Very Often

In the last month, how often have you found that you could not cope with all the things that you had to do?

Never

Almost Never

Sometimes

Fairly Often

Very Often

In the last month, how often have you been able to control irritations in your life?

Never

Almost Never

Sometimes

Fairly Often

Very Often

In the last month, how often have you felt that you were on top of things?

Never

Almost Never

Sometimes

Fairly Often

Very Often

In the last month, how often have you been angered because of things that happened that were outside of your control?

Never

Almost Never

Sometimes

Fairly Often

Very Often

In the last month, how often have you felt difficulties were piling up so high that you could not overcome them?

Never

Almost Never

Sometimes

Fairly Often

Very Often

Appendix E: System Usability Scale (SUS)

**Please answer the following questions thinking of Personalized Trials as the system:**

1. I think that I would like to use this system frequently:

1 – Strongly Disagree

2

3

4

5 – Strongly Agree

1. I found the system unnecessarily complex:

1 – Strongly Disagree

2

3

4

5 – Strongly Agree

1. I thought the system was easy to use:

1 – Strongly Disagree

2

3

4

5 – Strongly Agree

1. I think that I would need the support of a technical person to be able to use this system:

1 – Strongly Disagree

2

3

4

5 – Strongly Agree

1. I found the various functions in this system were well integrated:

1 – Strongly Disagree

2

3

4

5 – Strongly Agree

1. I thought there was too much inconsistency in this system:

1 – Strongly Disagree

2

3

4

5 – Strongly Agree

1. I would imagine that most people would learn to use this system very quickly:

1 – Strongly Disagree

2

3

4

5 – Strongly Agree

1. I found the system very awkward to use:

1 – Strongly Disagree

2

3

4

5 – Strongly Agree

1. I felt very confident using the system:

1 – Strongly Disagree

2

3

4

5 – Strongly Agree

1. I needed to learn a lot of things before I could get going with this system:

1 – Strongly Disagree

2

3

4

5 – Strongly Agree

Appendix F: Satisfaction Survey

**Please answer the following questions thinking back on your experience with Personalized Trials**

I found the onboarding process (from the initial survey to getting my materials) for my Personalized Trial straightforward and easy to follow:

1 – Strongly Disagree

2

3

4

5 – Strongly Agree

I think my Fitbit was easy to use:

1 – Strongly Disagree

2

3

4

5 – Strongly Agree

The informational videos helped me understand how to participate in this study:

1 – Strongly Disagree

2

3

4

5 – Strongly Agree

The materials I received were clear and easy to follow:

1 – Strongly Disagree

2

3

4

5 – Strongly Agree

I enjoyed receiving daily text message prompts and surveys on my cell phone:

1 – Strongly Disagree

2

3

4

5 – Strongly Agree

I felt like I knew what was coming next in my Personalized Trial:

1 – Strongly Disagree

2

3

4

5 – Strongly Agree

My Personalized Trial was easy to integrate into my daily routine:

1 – Strongly Disagree

2

3

4

5 – Strongly Agree

**Please answer the following questions about the participant report you received.**

The report explanation videos helped me to understand my report:

1 – Strongly Disagree

2

3

4

5 – Strongly Agree

I found the data report easy to understand:

1 – Strongly Disagree

2

3

4

5 – Strongly Agree

I found the data report useful for understanding how my stress affects me:

1 – Strongly Disagree

2

3

4

5 – Strongly Agree

I think this report will change the way I manage my stress in the future:

1 – Strongly Disagree

2

3

4

5 – Strongly Agree

I would need a conversation with someone to help me understand my report:

1 – Strongly Disagree

2

3

4

5 – Strongly Agree

**How satisfied were you with the following?**

Your Personalized Trial for Stress Management:

Not at all satisfied

A little satisfied

Somewhat satisfied

Very satisfied

Video explanations and demonstrations of study device and procedures:

Not at all satisfied

A little satisfied

Somewhat satisfied

Very satisfied

Text messaging for reminders:

Not at all satisfied

A little satisfied

Somewhat satisfied

Very satisfied

Text messaging for survey questions:

Not at all satisfied

A little satisfied

Somewhat satisfied

Very satisfied

Accessing the intervention videos:

Not at all satisfied

A little satisfied

Somewhat satisfied

Very satisfied

The yoga intervention video:

Not at all satisfied

A little satisfied

Somewhat satisfied

Very satisfied

The mindfulness meditation intervention video:

Not at all satisfied

A little satisfied

Somewhat satisfied

Very satisfied

The brisk walking intervention audio:

Not at all satisfied

A little satisfied

Somewhat satisfied

Very satisfied

Use of the Fitbit to track your activity and sleep:

Not at all satisfied

A little satisfied

Somewhat satisfied

Very satisfied

Use of the study’s communication over text message:

Not at all satisfied

A little satisfied

Somewhat satisfied

Very satisfied

Presentation of your results:

Not at all satisfied

A little satisfied

Somewhat satisfied

Very satisfied

**How much would you recommend this Personalized Trial for Stress Management to other persons with stress?**

I would not recommend

I would recommend a little bit

I would strongly recommend

**Overall, how helpful was your participation in this study with respect to your symptoms of stress?**

Not at all helpful

A little bit helpful

Somewhat helpful

Very much helpful

Extremely helpful

**Do you have any other comments or feedback about your experience participating in this Personalized Trial for Stress Management? If so, please use the text box provided below to share your thoughts.**

Appendix G: Standard Protocol Items: Recommendations for Interventional Trials (SPIRIT) Checklist


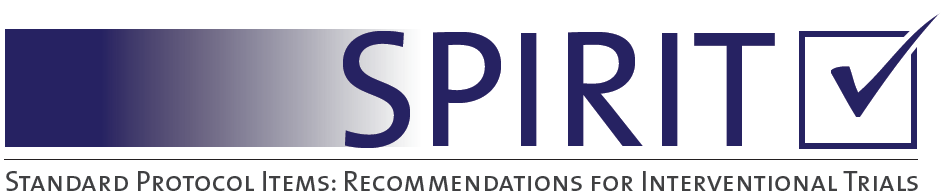


SPIRIT 2013 Checklist: Recommended items to address in a clinical trial protocol and related documents*

| **Section/item** | **ItemNo** | **Description** |
| --- | --- | --- |
| **Administrative information** | | |
| Title | 1 | Descriptive title identifying the study design, population, interventions, and, if applicable, trial acronym |
| Trial registration | 2a | Trial identifier and registry name. If not yet registered, name of intended registry |
|  | 2b | All items from the World Health Organization Trial Registration Data Set |
| Protocol version | 3 | Date and version identifier |
| Funding | 4 | Sources and types of financial, material, and other support |
| Roles and responsibilities | 5a | Names, affiliations, and roles of protocol contributors |
|  | 5b | Name and contact information for the trial sponsor |
|  | 5c | Role of study sponsor and funders, if any, in study design; collection, management, analysis, and interpretation of data; writing of the report; and the decision to submit the report for publication, including whether they will have ultimate authority over any of these activities |
|  | 5d | Composition, roles, and responsibilities of the coordinating centre, steering committee, endpoint adjudication committee, data management team, and other individuals or groups overseeing the trial, if applicable (see Item 21a for data monitoring committee) |
| **Introduction** |  |  |
| Background and rationale | 6a | Description of research question and justification for undertaking the trial, including summary of relevant studies (published and unpublished) examining benefits and harms for each intervention |
|  | 6b | Explanation for choice of comparators |
| Objectives | 7 | Specific objectives or hypotheses |
| Trial design | 8 | Description of trial design including type of trial (eg, parallel group, crossover, factorial, single group), allocation ratio, and framework (eg, superiority, equivalence, noninferiority, exploratory) |
| **Methods: Participants, interventions, and outcomes** | | |
| Study setting | 9 | Description of study settings (eg, community clinic, academic hospital) and list of countries where data will be collected. Reference to where list of study sites can be obtained |
| Eligibility criteria | 10 | Inclusion and exclusion criteria for participants. If applicable, eligibility criteria for study centres and individuals who will perform the interventions (eg, surgeons, psychotherapists) |
| Interventions | 11a | Interventions for each group with sufficient detail to allow replication, including how and when they will be administered |
|  | 11b | Criteria for discontinuing or modifying allocated interventions for a given trial participant (eg, drug dose change in response to harms, participant request, or improving/worsening disease) |
|  | 11c | Strategies to improve adherence to intervention protocols, and any procedures for monitoring adherence (eg, drug tablet return, laboratory tests) |
|  | 11d | Relevant concomitant care and interventions that are permitted or prohibited during the trial |
| Outcomes | 12 | Primary, secondary, and other outcomes, including the specific measurement variable (eg, systolic blood pressure), analysis metric (eg, change from baseline, final value, time to event), method of aggregation (eg, median, proportion), and time point for each outcome. Explanation of the clinical relevance of chosen efficacy and harm outcomes is strongly recommended |
| Participant timeline | 13 | Time schedule of enrolment, interventions (including any run-ins and washouts), assessments, and visits for participants. A schematic diagram is highly recommended (see Figure) |
| Sample size | 14 | Estimated number of participants needed to achieve study objectives and how it was determined, including clinical and statistical assumptions supporting any sample size calculations |
| Recruitment | 15 | Strategies for achieving adequate participant enrolment to reach target sample size |
| **Methods: Assignment of interventions (for controlled trials)** | | |
| Allocation: |  |  |
| Sequence generation | 16a | Method of generating the allocation sequence (eg, computer-generated random numbers), and list of any factors for stratification. To reduce predictability of a random sequence, details of any planned restriction (eg, blocking) should be provided in a separate document that is unavailable to those who enrol participants or assign interventions |
| Allocation concealment mechanism | 16b | Mechanism of implementing the allocation sequence (eg, central telephone; sequentially numbered, opaque, sealed envelopes), describing any steps to conceal the sequence until interventions are assigned |
| Implementation | 16c | Who will generate the allocation sequence, who will enrol participants, and who will assign participants to interventions |
| Blinding (masking) | 17a | Who will be blinded after assignment to interventions (eg, trial participants, care providers, outcome assessors, data analysts), and how |
|  | 17b | If blinded, circumstances under which unblinding is permissible, and procedure for revealing a participant’s allocated intervention during the trial |
| **Methods: Data collection, management, and analysis** | | |
| Data collection methods | 18a | Plans for assessment and collection of outcome, baseline, and other trial data, including any related processes to promote data quality (eg, duplicate measurements, training of assessors) and a description of study instruments (eg, questionnaires, laboratory tests) along with their reliability and validity, if known. Reference to where data collection forms can be found, if not in the protocol |
|  | 18b | Plans to promote participant retention and complete follow-up, including list of any outcome data to be collected for participants who discontinue or deviate from intervention protocols |
| Data management | 19 | Plans for data entry, coding, security, and storage, including any related processes to promote data quality (eg, double data entry; range checks for data values). Reference to where details of data management procedures can be found, if not in the protocol |
| Statistical methods | 20a | Statistical methods for analysing primary and secondary outcomes. Reference to where other details of the statistical analysis plan can be found, if not in the protocol |
|  | 20b | Methods for any additional analyses (eg, subgroup and adjusted analyses) |
|  | 20c | Definition of analysis population relating to protocol non-adherence (eg, as randomised analysis), and any statistical methods to handle missing data (eg, multiple imputation) |
| **Methods: Monitoring** | | |
| Data monitoring | 21a | Composition of data monitoring committee (DMC); summary of its role and reporting structure; statement of whether it is independent from the sponsor and competing interests; and reference to where further details about its charter can be found, if not in the protocol. Alternatively, an explanation of why a DMC is not needed |
|  | 21b | Description of any interim analyses and stopping guidelines, including who will have access to these interim results and make the final decision to terminate the trial |
| Harms | 22 | Plans for collecting, assessing, reporting, and managing solicited and spontaneously reported adverse events and other unintended effects of trial interventions or trial conduct |
| Auditing | 23 | Frequency and procedures for auditing trial conduct, if any, and whether the process will be independent from investigators and the sponsor |
| **Ethics and dissemination** | | |
| Research ethics approval | 24 | Plans for seeking research ethics committee/institutional review board (REC/IRB) approval |
| Protocol amendments | 25 | Plans for communicating important protocol modifications (eg, changes to eligibility criteria, outcomes, analyses) to relevant parties (eg, investigators, REC/IRBs, trial participants, trial registries, journals, regulators) |
| Consent or assent | 26a | Who will obtain informed consent or assent from potential trial participants or authorised surrogates, and how (see Item 32) |
|  | 26b | Additional consent provisions for collection and use of participant data and biological specimens in ancillary studies, if applicable |
| Confidentiality | 27 | How personal information about potential and enrolled participants will be collected, shared, and maintained in order to protect confidentiality before, during, and after the trial |
| Declaration of interests | 28 | Financial and other competing interests for principal investigators for the overall trial and each study site |
| Access to data | 29 | Statement of who will have access to the final trial dataset, and disclosure of contractual agreements that limit such access for investigators |
| Ancillary and post-trial care | 30 | Provisions, if any, for ancillary and post-trial care, and for compensation to those who suffer harm from trial participation |
| Dissemination policy | 31a | Plans for investigators and sponsor to communicate trial results to participants, healthcare professionals, the public, and other relevant groups (eg, via publication, reporting in results databases, or other data sharing arrangements), including any publication restrictions |
|  | 31b | Authorship eligibility guidelines and any intended use of professional writers |
|  | 31c | Plans, if any, for granting public access to the full protocol, participant-level dataset, and statistical code |
| **Appendices** |  |  |
| Informed consent materials | 32 | Model consent form and other related documentation given to participants and authorised surrogates |
| Biological specimens | 33 | Plans for collection, laboratory evaluation, and storage of biological specimens for genetic or molecular analysis in the current trial and for future use in ancillary studies, if applicable |

*It is strongly recommended that this checklist be read in conjunction with the SPIRIT 2013 Explanation & Elaboration for important clarification on the items. Amendments to the protocol should be tracked and dated. The SPIRIT checklist is copyrighted by the SPIRIT Group under the Creative Commons “[Attribution-NonCommercial-NoDerivs 3.0 Unported](http://www.creativecommons.org/licenses/by-nc-nd/3.0/)” license.
